# Developmental excitation-inhibition imbalance underlying psychoses revealed by single-cell analyses of discordant twins-derived cerebral organoids

**DOI:** 10.1038/s41380-020-0844-z

**Published:** 2020-08-07

**Authors:** Tomoyo Sawada, Thomas E. Chater, Yohei Sasagawa, Mika Yoshimura, Noriko Fujimori-Tonou, Kaori Tanaka, Kynon J. M. Benjamin, Apuã C. M. Paquola, Jennifer A. Erwin, Yukiko Goda, Itoshi Nikaido, Tadafumi Kato

**Affiliations:** 1 Laboratory for Molecular Dynamics of Mental Disorders, RIKEN Center for Brain Science, Wako, Saitama Japan; 2 Lieber Institute for Brain Development, Baltimore, MD USA; 3 Department of Neurology, Johns Hopkins University School of Medicine, Baltimore, MD USA; 4 Laboratory for Synaptic Plasticity and Connectivity, RIKEN Center for Brain Science, Wako, Saitama Japan; 5 Laboratory for Bioinformatics Research, RIKEN Center for Biosystems Dynamics Research, Wako, Saitama Japan; 6 Department of Neuroscience, Johns Hopkins School of Medicine, Baltimore, MD USA; 7 Functional Genome Informatics, Medical Research Institute, Tokyo Medical and Dental University, Bunkyo, Tokyo Japan; 8 Master’s/Doctoral Program in Life Science Innovation (Bioinformatics), Degree Programs in Systems and Information Engineering, Graduate School of Science and Technology, University of Tsukuba, Tsukuba, Ibaraki Japan; 9 Department of Psychiatry and Behavioral Science, Juntendo University Graduate School of Medicine, Bunkyo, Tokyo Japan

**Keywords:** Stem cells, Schizophrenia, Molecular biology, Neuroscience, Bipolar disorder

## Abstract

Despite extensive genetic and neuroimaging studies, detailed cellular mechanisms underlying schizophrenia and bipolar disorder remain poorly understood. Recent progress in single-cell RNA sequencing (scRNA-seq) technologies enables identification of cell-type-specific pathophysiology. However, its application to psychiatric disorders is challenging because of methodological difficulties in analyzing human brains and the confounds due to a lifetime of illness. Brain organoids derived from induced pluripotent stem cells (iPSCs) of the patients are a powerful avenue to investigate the pathophysiological processes. Here, we generated iPSC-derived cerebral organoids from monozygotic twins discordant for psychosis. scRNA-seq analysis of the organoids revealed enhanced GABAergic specification and reduced cell proliferation following diminished Wnt signaling in the patient, which was confirmed in iPSC-derived forebrain neuronal cells. Two additional monozygotic twin pairs discordant for schizophrenia also confirmed the excess GABAergic specification of the patients’ neural progenitor cells. With a well-controlled genetic background, our data suggest that unbalanced specification of excitatory and inhibitory neurons during cortical development underlies psychoses.

## Introduction

Schizophrenia (SZ) and bipolar disorder (BD) are among the most intractable disorders in brain health. Both illnesses share complex genetic/environmental etiologies, molecular neuropathology, neurodevelopmental origin, and a subset set of symptoms including psychosis. Genetic and neuroimaging studies have shown clues to understand biological bases of these psychiatric disorders. However, responsible cell types for the pathogenesis and consequences of their dysfunction have not been fully elucidated because of methodological limitations on cellular complexity of the brain.

Recent development and progress of single-cell RNA sequencing (scRNA-seq) technologies enabled the identification of cell-type-specific pathological processes. Although scRNA-seq has been applied to human postmortem brains [1–3], confounding factors and methodological difficulties hamper the application of this technology to identification of cellular alterations underlying psychiatric disorders with patients’ postmortem brains.

Brain organoids have become an important experimental tool to investigate structural and functional complexity of human brain development and neurodevelopmental disorders [4–7]. Taking neurodevelopmental origins into account, brain organoids derived from induced pluripotent stem cells (iPSCs) of the patients are an optimal tool to identify the cell-type-specific pathophysiology of SZ and BD.

Mounting research using patient-derived iPSCs has proposed several cellular pathways altered in these psychiatric disorders, including synaptic function and Wnt signaling [8]. However, most of these studies differentiated the iPSCs into a predefined specific cell type. Therefore, the developmental trajectory that leads to altered brain development in these disorders remains unknown. Moreover, sporadic case-control designs used in the previous studies have large heterogeneous genotypic differences. Due to substantial heterogeneity, the specificity of and relationships among these pathways in neuropsychiatric disorders and the developmental trajectory that leads to altered brain construction remain unclear. Thus, simulating brain development with a well-controlled genetic background [9] is also essential for progress in this field.

Here, we applied Quartz-Seq2 [10], which showed outstanding performance among scRNA-seq methods by benchmarking [11], to iPSC-derived cerebral organoid models of psychosis. To detect disease-specific alterations minimizing inter-individual genetic variations, we analyzed the organoids of a pair of monozygotic twins discordant for psychoses and verified the findings in two additional monozygotic twins discordant for SZ.

## Materials and methods

### Participants

**Fig. 1:**
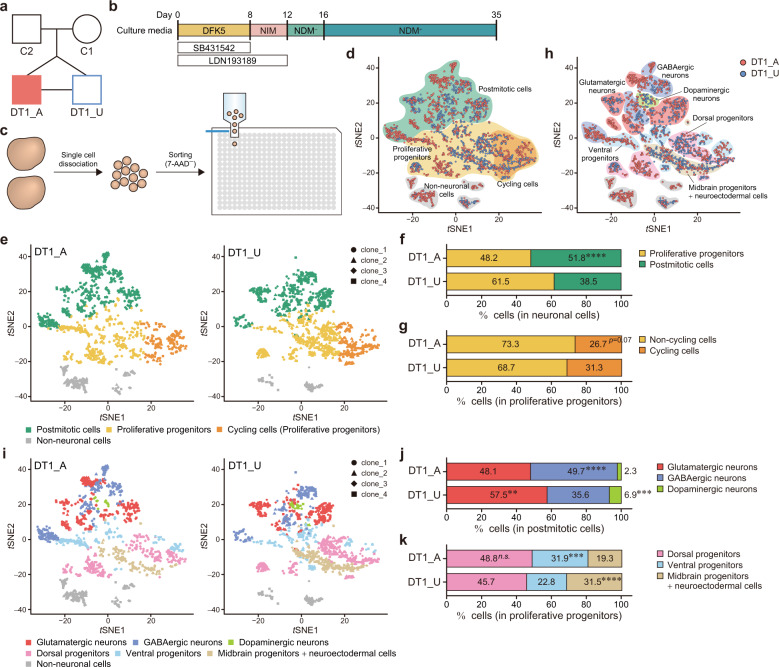
scRNA-seq shows accelerated neuronal differentiation and enhanced GABAergic specification in cerebral organoids derived from the psychosis-affected twin. **a** Diagram of the pedigree, including a pair of monozygotic twins discordant for SA-B. DT1_A, affected twin; DT1_U, unaffected twin; C1, mother; C2, father. **b** Schematic protocol for the generation of cerebral organoids. **c** Overview of sample preparation for scRNA-seq. The 7-AAD^−^ live single cells dissociated from two organoids of each iPSC clone were sorted into 384-well plates containing cell lysis buffer with barcoding primers. **d** *t*-distributed stochastic neighbor embedding (*t*SNE) plot according to cellular maturity based on the expression patterns of marker genes. **e** *t*SNE plots based on cellular maturity. Data from DT1_A and DT1_U are shown separately. Proportion of proliferative progenitors and postmitotic cells among the neuronal population (**f**) and of non-cycling cells and cycling cells among proliferative progenitors (**g**). **h** *t*SNE plot according to neuronal subtypes based on the expression patterns of marker genes. **i** *t*SNE plots based on the neuronal subtypes. Data from DT1_A and DT1_U are shown separately. The breakdown of postmitotic cells (**j**) and proliferative progenitors (**k**). Fisher’s exact test: **p < 0.01; ***p < 0.001; ****p < 0.0001; n.s. not significant (**f**, **g**, **j** and **k**). See also Figs. S2–4.

A Japanese family including a pair of male monozygotic twins discordant for schizoaffective disorder, bipolar type (DT1_A, DT1_U, C1, and C2) (Figs. 1a and S1A) and two pairs of monozygotic twins discordant for SZ (DT2_A and DT2_U [12] and DT3_A and DT3_U [13] (Fig. S1A) were included in this study. Array-based comparative genome hybridization (array CGH) analysis confirmed monozygosity of DT1 (Fig. S1B). All twins had been discordant for the disease for at least 3 years prior to the day of blood sampling. Two of healthy male control individuals (C3 and C4) (Fig. S1A) were also enrolled. All participants were screened for mental disorders by trained psychiatrists using the Mini International Neuropsychiatric Interview [14]. Detailed information regarding participant characteristics is presented in Fig. S1A. Written informed consent was obtained from all participants, and all experiments involving human iPSCs were approved by the Wako 1st Research Ethics Committee of RIKEN (Wako 1: 15-4(41)).

### Derivation, cultivation, and characterization of human iPSCs

For DT1_A, DT1_U, C1, C2, C3, and C4, iPSCs were established from peripheral blood mononuclear cells (PBMCs). PBMCs were purified via density gradient centrifugation with Ficoll-Paque PREMIUM (GE Healthcare, IL, USA), in accordance with the manufacturer’s instructions. We generated iPSCs using episomal vectors, as previously described [15, 16]. Briefly, plasmids pCXLE-hOCT3/4-shp53-F, pCXLE-hSK, pCXLE-hUL, and pCXWB-EBNA1 (Addgene, MA, USA) were transfected into PBMCs using Nucleofector II (Lonza, Basel, Switzerland; program V-024) at a concentration of 3 μg per 100 μl solution per 3 × 10^6^ cells, and an Amaxa Human T Cell Nucleofector kit (Lonza). The transfected cells were then seeded onto 100-mm dishes covered with mitomycin C-treated SNL76/7 feeder cells (ATCC, VA, USA) in KBM502 (Kohjin Bio, Saitama, Japan) supplemented with 16.7 μl/10^6^ cells of Dynabeads Human T-Activator CD3/CD28 (Thermo Fisher Scientific, MA, USA). Two days after transfection, an equal volume of iPSC medium consisting of DMEM/F12 + GlutaMAX, 20% Knockout Serum Replacement (KSR), 1 × MEM NEAA, 100 μM 2-melcaptoethanol, 0.5 × penicillin/streptomycin (Thermo Fisher Scientific), and 4 ng/ml human basic FGF (bFGF) (Wako, Osaka, Japan) was added. The culture medium was then replaced with iPSC medium every other day until iPSC colonies appeared. For DT2_A, DT2_U, DT3_A, and DT3_U, iPSCs were generated from lymphoblastoid cell lines (LCLs). LCLs were maintained in LCL medium consisting of RPMI 1640 medium (Thermo Fisher Scientific) supplemented with 10% FBS and 0.5 × penicillin/streptomycin. In accordance with previously described methods [17], cells were reprogrammed by electroporating 2 × 10^6^ cells with 1 μg each of pCXLE-hOCT3/4-shp53-F, pCXLE-hSK, and pCXLE-hUL using a Neon transfection system (Thermo Fisher Scientific). Transfection parameters were as follows: 1100 V, 30 ms, two pulses. Transfected cells were cultivated in the LCL medium for 24 h, following which half of the medium was replaced with iPSC medium and were further incubated for 24 h. Forty-eight hours after electroporation, the cells were seeded onto SNL feeder cells and maintained in iPSC medium. Three to five weeks later, iPSC colonies were manually picked up for further expansion and characterization. The iPSCs were maintained on feeder cells in iPSC medium and passaged every 4–6 days with CTK solution consisting of 0.25% trypsin, 0.1 mg/ml Collagenase Type IV, 1 mM CaCl_2_, and 20% KSR as previously described [18].

To evaluate a neural differentiation potency of established iPSCs, we differentiated them (eight clones each from DT1_A and DT1_U) into neural progenitor cells (NPCs) via the serum-free floating culture of embryoid body-like aggregates with quick reaggregation (SFEBq) method, as previously described [19] (Fig. S1C). Briefly, iPSCs treated overnight with 10 μM Y-27632 (Wako) were dissociated into single cells using Accumax (Innovative Cell Technologies, CA, USA) and transferred to PrimeSurface 96U plates (Sumitomo bakelite, Tokyo, Japan) at a density of 9000 cells/well. The cells were cultured for 14 days in DFK5 medium consisting of DMEM/F12 + GlutaMAX, 5% KSR, 1 × MEM NEAA, 100 μM 2-ME, and 0.5 × penicillin/streptomycin. The medium was supplemented with 30 μM Y-27632, 10 μM SB431542 (Sigma, MO, USA), and 100 nM LDN-193189 (Miltenyi Biotec, Bergisch Gladbach, Germany) for the first 4 days. Neural aggregates on day 14 were dissociated into single cells using Accutase (Innovative Cell Technologies) and stained with anti-PSA-NCAM-PE (Miltenyi Biotec) and anti-TRA-1-60-FITC (BD Biosciences, NJ, USA) antibodies, in accordance with the manufacturer’s protocols. The cells were then stained with 7-AAD (BD Biosciences) to exclude dead cells and analyzed using FACS Aria II (BD Biosciences). Unstained cells were used as a negative control. Data were analyzed using FlowJo software (BD Biosciences). Expression of pluripotent stem cell markers was examined by quantitative RT-PCR (RT-qPCR) and RT-PCR with primers listed on Table S1 and immunofluorescent staining with antibodies against OCT4, TRA-1-60, and TRA-1-81 (see details in Table S2). The pluripotency of established iPSC lines was validated by in vitro spontaneous differentiation assay. Cell clumps of iPSCs harvested with CTK solution were transferred to 100-mm Ultra Low Attachment Culture Dishes (Corning, NY, USA) in iPSC medium without bFGF. The medium was changed every other day. Twenty-day-old embryoid bodies (EBs) were harvested and total RNA was extracted. The expression of three germ layer markers was analyzed via RT-PCR with primers listed on Table S1. To confirm the absence of genomic integration of episomal vectors utilized for reprogramming, we performed genomic PCR using primers for the OriP cassette (Table S1) with Ex Taq HS (Takara Bio, Shiga, Japan). Karyotyping analysis of the iPSCs was conducted via G-banding (Chromocenter Inc., Tottori, Japan).

### Cell culture

SNL76/7 feeder cells were maintained in DMEM (Thermo Fisher Scientific) supplemented with 10% FBS and 0.5 × penicillin/streptomycin on 0.1% gelatin-coated plates. To inhibit proliferation, we treated the SNL76/7 cells with 12.4 μg/ml Mitomycin C (Sigma) for 2.25 h. The human iPSC line 409B2 was provided by the RIKEN BRC (Ibaraki, Japan) through the Project for Realization of Regenerative Medicine and the National Bio-Resource Project of the MEXT, Japan [15]. Human dermal fibroblasts (HDFs) (RIKEN BRC) were maintained in MEM α (Thermo Fisher Scientific) supplemented with 10% FBS. To quantify the exogenous expression of reprogramming factors, HDFs were transfected with the episomal vectors using Neon at a concentration of 3 μg per 3 × 10^5^ cells. Transfection parameters were as follows: 1650 V, 10 ms, three pulses. Four days after electroporation, the cells were harvested and subjected to further analyses. For RT-PCR, PBMCs were expanded in KBM502 on 100-mm dishes coated with anti-Human CD3 antibodies (BD Biosciences) to activate the proliferation of T cells.

### Genomic DNA extraction

Genomic DNA was extracted from peripheral blood samples using a genomic DNA Extraction kit (Genomix, Trieste, Italy). Genomic DNA of cultured cells was extracted using a DNeasy Blood & Tissue kit (Qiagen, Hilden, Germany). The concentration of extracted dsDNA was measured using a Qubit 2.0 Fluorometer and a Qubit dsDNA HS Assay kit (Thermo Fisher Scientific).

### RNA extraction and (q)PCR

Total cellular RNA was extracted from iPSCs or neuronal cells using TRIzol Reagent (Thermo Fisher Scientific) and a Direct-zol RNA MiniPrep kit (Zymo Research, CA, USA), in accordance with the manufacturer’s instructions. Reverse transcription was performed with the oligo dT20 primer using a RverTra Ace-α-kit (Toyobo, Osaka, Japan). RT-PCR was carried out with Ex Taq HS on Veriti 96-Well Thermal Cycler (Thermo Fisher Scientific). RT-qPCR was performed using SYBR Premix Ex Taq II (Takara Bio) on QuantStudio 12 K Flex Real-Time PCR System (Thermo Fisher Scientific). The primer sequences are shown in Table S1.

### Array-based comparative genome hybridization (array CGH) analysis

Array CGH was performed using a SurePrint G3 Human CGH Microarray kit, 1 × 1 M (Agilent Technologies, CA, USA). Labeling was performed using a SureTag Complete DNA Labeling kit (Agilent Technologies). Microarray slides were scanned using an Agilent microarray scanner, and scanned images were digitized using Feature Extraction Software. The data were then analyzed using GeneSpring GX (Agilent Technologies).

### Generation and culture of cerebral organoids

Cerebral organoids were generated as previously described, with modifications [20]. Briefly, after removal of SNL feeders from iPSC cultures with CTK solution, iPSCs were dissociated into single cells using Accumax and transferred to PrimeSurface 96U plates at a density of 9000 cells/well. The cells were cultured for 8 days in DFK5 medium supplemented with 10 μM SB431542 and 100 nM LDN-193189. The medium was also supplemented with 30 μM Y-27632 for the first 4 days. On day 8, EBs were transferred to Ultra Low Attachment 24-well plates (Corning) in neural induction medium (NIM) consisting of DMEM/F12 + GlutaMAX, 1 × N2 Supplement (Thermo Fisher Scientific), 1 × MEM NEAA, 1 μg/ml Heparin (Sigma), 0.5 × penicillin/streptomycin, and 100 nM LDN-193189. An equal volume of NIM was added 48 h after transferring them to 24-well plates. On day 12, each EB was embedded in a Matrigel Basement Membrane Matrix Growth Factor Reduced (GFR) (Corning) droplet and transferred to a 60-mm culture dish in neural differentiation medium minus (NDM^−^) consisting of a 1:1 mixture of DMEM/F12 and Neurobasal medium (Thermo Fisher Scientific) supplemented with 0.5 × N2 Supplement, 0.5 × B27 Supplement minus Vitamin A (Thermo Fisher Scientific), 0.5 × MEM NEAA, 1 × GlutaMAX, 100 μM 2-ME, and 2.65 μg/ml Insulin without agitation. The medium was replaced on day 14. After 4 days in static culture, the medium was changed to neural differentiation medium plus (NDM^+^) in which B27 Supplement minus Vitamin A was replaced with B27 Supplement (with Vitamin A). The culture was then agitated on an orbital shaker (IKA, Staufen, Germany) at 80 r.p.m. The medium was replaced every 3–4 days. For rescue experiments, the EBs/organoids were treated with 1 mM LiCl (Sigma) or 100 ng/ml Wnt3a (R&D systems, MN, USA) for 8 days (days 4–12). On days 30 and 35, the organoids were fixed and immunostained with appropriate antibodies, and subjected to scRNA-seq analysis, respectively. The size of the SOX2^+^ ventricular zone (VZ)-like layer was measured using ImageJ software [21]. The quantification was performed in an unblinded manner. The ambiguous boundary between SOX2^+^ and SOX2^−^ cells was determined based on the expression of DCX, which was co-stained with SOX2 on the serial sections. The Fluorescence intensity of GABA on the cerebral organoid slices were measured with ImageJ software. Threshold was determined by Yen’s methods. Total fluorescence intensity was divided by the area.

### Quartz-Seq2 single-cell RNA-seq analysis

On day 35, cerebral organoids from DT1_A and DT1_U (two organoids for each iPSC clone) were incubated in cell recovery solution (Corning) on ice for 45 min. After washing twice with PBS, the organoids were incubated in Accutase at 37 °C for 20 min and then dissociated into single cells by pipetting up and down. Cells were rinsed with FACS buffer consisting of HBSS (Thermo Fisher Scientific) supplemented with 0.5% BSA (MP Biomedicals, CA, USA) and filtered through a 35-µm nylon mesh (BD Biosciences). After centrifugation, cells were resuspended in FACS buffer and stained with 7-AAD at room temperature for 15 min. Each living single cell was then sorted into lysis buffer in a 384-well PCR plate (Eppendorf, Hamburg, Germany) at 4 °C using FACS Aria II. Sequence library DNA was prepared in accordance with methods described in our previous study [10]. The libraries were analyzed using NextSeq 500/550 High Output v2 kit (75 cycles) with NextSeq 500 sequencer (Illumina, CA, USA). The sequence specification of Quartz-Seq2 was as follows: Read1, 22 cycles; index1, 6 cycles; and Read2, 64 cycles. Bcl files were converted to fastq files using bcl2fastq2 (v2.17.1.14) (Illumina). We then processed fastq files into a digital expression matrix, as in our previous study [10]. During this process, we trimmed read2 length to 63 nucleotides using the FASTX-Toolkit (version 0.0.14) (https://github.com/agordon/fastx_toolkit/blob/master/reconf). The versions of databases for the process were as follows: human genome, GRCh38.primary assembly.genome.fa; genome annotation, gencode.v27.primary_assembly.annotation.gtf; and Drop-seq tools, v1.11 [22]. The average number of fastq reads per well was ~95,000. We detected ~4800 gene counts and 20,000 unique molecular identifier counts per well on average. The copy number of ERCC spike-in RNA at 50% detection probability for Quartz-Seq2 was ~2.18 (Table S3).

Highly variable genes across a population of cells were selected for secondary analysis using DESeq2 1.16.1 [23] and statmod 1.4.30 R package [24]. First, we selected genes with CV2 values higher than 0.3, following which we calculated the 95th percentile. We then removed genes with median values below the 95th percentile, and the average expression in each gene and log-transformed CV2 were fitted using the glmgam.fit function. Finally, we evaluated the statistical significance of the deviation from the fitting curve for each gene using chi-square tests, setting the *p*-value parameter to 0.001 (final set: 7469 genes). Abnormal cell clusters with a proportion of mitochondrial gene expression higher than 12% or fewer than 10–3000 expressed genes were excluded using the kmeans function in stats R package (https://stat.ethz.ch/R-manual/R-devel/library/stats/html/00Index.html), setting the *k* parameter to 7 (final set: 2782 cells). The digital gene expression matrix was added to 1 and log-transformed. Dimensional reduction of cells derived from all organoids was performed using the Rtsne 0.13 function [25], setting the perplexity to 40. Cell typing was performed using kmeans based on the expression profile of cell-type-specific markers (Table S4) [26–29]. DEGs between affected and unaffected samples in each cell type were calculated using the glmLR function in edgeR 3.18.1 R package [30]. Pathway enrichment analysis of DEGs was performed using IPA software (Qiagen). For visualization of gene expression data, ggplot2 2.2.1 [31], pHeatmap 1.0.10 (https://cran.r-project.org/web/packages/pheatmap/index.html), and Rtsne 0.13 were used.

### Differentiation of iPSCs into forebrain-specific neural stem/progenitor cells (NSPCs) and cortical neurons

We differentiated iPSCs into forebrain-specific NSPCs according to a previous report [32] with slight modifications. On day 8 of cerebral organoid differentiation, EBs were transferred onto 6-well culture plates (Corning) coated with Matrigel GFR at a density of 48 EBs/well in DFN2 medium consisting of DMEM/F12 + GlutaMAX, 1 × N2 Supplement, 1 × MEM NEAA, 100 μM 2-ME, 0.5 × penicillin/streptomycin, and 100 nM LDN-193189. The medium was replaced every 2 days. On day 24, the cells were dissociated into single cells using Accutase and seeded at 150–250 cells/well in 24-well culture plates on a bed of neonatal murine cortical astrocytes grown on 10 mm glass cover slips or 13.5 mm Celldesk LF1 (Sumitomo bakelite) in NB27 medium consisting of Neurobasal medium, 1 × GlutaMAX-I, B27 Supplement minus Vitamin A, and 0.5 × penicillin/streptomycin. The medium was supplemented with 10 μM Y-27632 from days 24 to 28. Half of the medium was replaced every 4 days during continuous culturing. For rescue experiments, cells were treated with 1 mM LiCl or 100 ng/ml human Wnt3a from days 4 to 24. Cell viability was examined via flow cytometry on day 8. The cells were dissociated into single cells using Accutase, labeled by 7-AAD, and then analyzed using FACS Aria II. Unstained cells were used as a negative control. Data were analyzed using FlowJo software.

### EdU incorporation assay

To measure the proliferative activity of NSPCs, EdU incorporation assays were performed in 8-day-old EBs and 30-day-old NSPCs. For EB samples, cells were incubated with 10 μM EdU for 3 or 24 h. Thereafter, cells were dissociated into single cells using Accutase and stained with anti-CD15-APC antibody (BD Biosciences), in accordance with the manufacturer’s instructions. Then, the cells were prepared for detection using a Click-iT Plus EdU Flow Cytometry Assay kit (Thermo Fisher Scientific), in accordance with the manufacturer’s protocol. DNA was stained with 1 μg/ml DAPI (BD Biosciences). The cells were analyzed using FACS Aria II. Unstained cells were used as a negative control. Data were analyzed using FlowJo software. For 30-day-old NSPCs, cells were labeled with 10 μM EdU for 3 h, and signal detection was performed using a Click-iT EdU Imaging kit (Thermo Fisher Scientific), according to the manufacturer’s protocol. After detecting EdU, cells were further immunostained with anti-Nestin antibody (MBL, Aichi, Japan). The proportion of EdU^+^/Nestin^+^ cells was measured using the ImageJ Cell Counter plugin.

### Electrophysiological analysis

Whole-cell patch-clamp recordings were obtained from iPSC-derived cortical neurons placed on the stage of an Axio Examiner upright microscope (Carl Zeiss, Oberkochen, Germany) using a Multiclamp amplifier (Molecular Devices, CA, USA). The recording chamber was continuously perfused with an extracellular solution containing 130 mM NaCl, 2.5 mM KCl, 2.2 mM CaCl_2_, 1.5 mM MgCl_2_, 10 mM D-glucose, and 10 mM HEPES (pH 7.35, osmolarity adjusted to 290 mOsm). For miniature IPSCs, 1 μM TTX (Tocris Bioscience, Bristol, UK) was added to the recording solution. For miniature EPSCs, 1 μM TTX and 50 μM picrotoxin (Sigma) were added to the recording solution. Micropipettes were made from borosilicate glass capillaries, with a resistance in the range of 3–6 MΩ. The intracellular solution contained 100 mM K-gluconate, 17 mM KCl, 5 mM NaCl, 5 mM MgCl_2_, 10 mM HEPES, 0.5 mM EGTA, 4 mM ATPK2, and 0.5 mM GTPNa (pH 7.3, osmolarity adjusted to 280 mOsm). All recordings were performed at room temperature (21–24 °C). Events were recorded at 10 kHz and analyzed offline. All analyses were performed using Clampfit software (Molecular Devices).

### Morphological analysis of iPSC-derived neurons

To assess the morphology of iPSC-derived cortical neurons, cells were transfected with a plasmid coding for EGFP (pEGFP-N1) (Clontech, CA, USA) using Lipofectamine 2000 (Thermo Fisher Scientific). Forty-eight hours after transfection, robust EGFP expression was visible, and cells were fixed in 4% paraformaldehyde (PFA)/sucrose in PBS for 15 min. Following permeabilization, cells were incubated with an anti-GFP antibody (Millipore, MA, USA) overnight at 4 °C in the presence of 0.3% Triton X-100 and 0.3% goat serum albumin. A secondary antibody diluted in incubation buffer was applied for 2 h at room temperature. Cells were imaged on a Zeiss 780 Axio Examiner (Carl Zeiss) using ×20 or ×63 objectives. For dendritic protrusion density analysis, Z-stacks were captured with a 0.3 μm slice depth, collapsed into maximum intensity projections, and protrusions were counted manually.

### Bulk RNA-seq analysis

NSPCs on day 8 and 24 were used for gene expression analyses. Total RNA was isolated using the TRIzol reagent and a PureLink RNA Micro kit (Thermo Fisher Scientific) or Direct-zol RNA MiniPrep kit, in accordance with the manufacturer’s instructions. Confirmation of the RNA quality was performed using an Agilent 2100 Bioanalyzer (Agilent Technologies). RNA-seq libraries were prepared from the purified RNA using a TruSeq RNA Sample Preparation kit v2 (Illumina). The length of libraries was 316–429 bp, including adapter sequences. Samples were sequenced paired end 2 × 101 bp on a HiSeq 2500 system (Illumina). For data processing, we aligned reads to the GRCh38 human genome (GENCODE release 26, GRCh38.p10) using HISAT2 (v2.1.0) [33]. We generated gene counts using the SubRead utility featureCounts (v1.5.3) [34] for paired end, reversed stranded read counting using read-level filters including: (1) uniquely mapped reads corresponding to a mapping quality of 60 for Hisat2 BAM files, (2) reads aligned in proper pairs, (3) excluded chimeric reads, and (4) primary alignment [=featureCounts -p -s 2 -Q 60 -B -C --primary -a $GTF -G $FASTA -o $OUTPUT $BAM = ]. Gene counts were converted to TPM using effective read lengths determined by using picard tools utility =CollectInsertSizeMetrics= to calculated mean insert size for all samples. Pathway analyses of DEGs were performed using IPA software.

### Immunocytochemistry

Cells were fixed with 4% PFA in PBS for 10 min at room temperature and then permeabilized with 0.2% Triton X-100 in PBS for 15 min at room temperature. Cells were then blocked with 10% normal donkey serum in PBS containing 0.01% Tween-20 for 30 min at room temperature or overnight at 4 °C. After blocking, cells were incubated with primary antibodies diluted in blocking solution at 4 °C overnight, followed which they were incubated with secondary antibodies for 2 h at room temperature. Cells were finally incubated with 1 μg/ml Hoechst 33342 (Thermo Fisher Scientific) for 10 min at room temperature. Fluorescent signals were detected using a Zeiss AxioObserver.Z1 Microscope with ApoTome.2 (Carl Zeiss), and images were processed using ZEN software (Carl Zeiss) and Photoshop CS6 software (Adobe, CA, USA). The numbers of excitatory and inhibitory synaptic puncta were manually counted. The size of Gephyrin puncta was analyzed using the ImageJ Analyze particles plugin. The proportion of GABAergic neurons was measured using the ImageJ Cell Counter plugin.

For immunostaining of cerebral organoids, the organoids were fixed with 4% PFA in PBS overnight at 4 °C on day 30. After washing with PBS, organoids were placed in serial dilutions of PBS-buffered sucrose (10, 20, and 30%, in sequence) at 4 °C. Each solution was replaced every day. The dehydrated organoids were maintained in 30% sucrose solution at 4 °C until embedding with OCT compound (Sakura Finetek, Tokyo, Japan). One day before cryosectioning, organoids were placed in a 1:2 mixture of 30% sucrose solution and OCT compound and left overnight at 4 °C. Then, organoids were embedded in a 1:2 mixture of 30% sucrose solution and OCT compound and frozen immediately in dry ice/acetone. Cryosections (10 μm) were subjected to additional fixation with 4% PFA in PBS for 3 min at room temperature, following which they were permeabilized and blocked with 10% normal donkey serum in PBS containing 0.3% Triton X-100 for 30 min at room temperature. After washing with 5% serum in PBS containing 0.01% Tween-20, sections were incubated with primary and secondary antibodies. Detailed information regarding antibodies is available on Table S2.

### Analysis of postmortem brain gene expression data

We downloaded BrainSeq Consortium, dorsolateral prefrontal cortex FASTQ files [35]. The reads were aligned as previously described [35, 36] with slight modification. Briefly, we generated TPM estimates by aligning the reads to the GRCh38 human genome (GENCODE release 26, GRCh38.p10) using Kallisto (v0.43.1) [37] and the reference transcriptome. The expression levels of transcripts encoding 146 genes annotated with GABAergic synapse and/or GABA receptor signaling were compared between the patients with SZ or schizoaffective disorder (“Schizo”) and non-psychiatric control individuals. Among multiple transcripts for each gene, we extracted one protein-coding transcript exhibited the highest expression in control samples.

### Statistical analysis

Statistical analyses were performed using GraphPad Prism 7 software (GraphPad Software, CA, USA) and SPSS software (IBM, NY, USA). Two- or one-tailed Student’s or Welch’s *t* tests were used for comparisons between two groups, while one-way ANOVA followed by Tukey’s multiple comparison tests were used for comparisons among three groups. To evaluate the effect of lithium chloride (LiCl) treatment, we performed two-way ANOVA with post hoc Student’s or Welch’s *t* tests. To evaluate the effect of clonal variation and LiCl treatment, we performed three-way ANOVA. We also used Fisher’s exact tests to compare the cell proportion in cerebral organoids between the twins in scRNA-seq analysis and for the expression patterns of GABAergic synapse genes in bulk RNA-sea and postmortem brains. No statistical method was used to predetermine the sample size. The sample sizes and the numbers of trials are indicated in Table S5. Detailed statistical information can be found in the figure legends and is shown in Table S6. The sample sizes and the number of trials are indicated in Table S7. Each graph was drawn using ggplot2 library [31] in R studio [38].

## Results

### Affected twin-derived cerebral organoids show accelerated neuronal differentiation and enhanced GABAergic specification

We first established iPSCs from a pair of male monozygotic twins (DT1) discordant for schizoaffective disorder, bipolar type (SA-B), which shares clinical features including psychosis with both SZ and BD (Figs. 1a and S1A, B). First, we screened the neuronal differentiation potency of the iPSCs from the twins (Fig. S1C–F) and validated the quality of multiple selected clones from each individual (affected twin: DT1_A1, 2, 3, 4 and unaffected twin: DT1_U1, 2, 3, 4) for further functional analyses (Fig. S1G–M). To investigate the neurodevelopmental alterations underlying psychosis, we differentiated iPSCs into cerebral organoids (Fig. 1b) [6, 20]. On day 35, we performed scRNA-seq analysis with the Quartz-Seq2 method (Fig. 1c) [10]. We first classified each cell into neuronal and non-neuronal populations based on patterns of marker gene expression (Figs. 1d, S2 and S3A–D, and Table S4). The neuronal population consisted of postmitotic cells expressing markers such as *MAPT* or *STMN2*, and proliferative progenitors were positive for markers such as *SOX2* or *NES* (Fig. 1d and Table S4). The non-neuronal population included progenitors for astroglia, oligodendrocytes, mesenchymal cells, and the choroid plexus (Table S4). The cerebral organoid from the psychotic twin (DT1_A) had significantly fewer proliferative progenitors and remarkably more postmitotic cells than the healthy co-twin (DT1_U)-derived organoids (Figs. 1e, f and S3E, F), implying an accelerated commitment to neuronal differentiation in the affected twin. Moreover, DT1_A organoids tended to have fewer cycling cells, expressing markers such as *HMGB2* or *MKI67* (Fig. 1d and Table S4), than DT1_U organoids among the proliferative progenitor population (Figs. 1e, g and S3E,G). We then further deconstructed neuronal cell composition according to gene expression patterns (Figs. 1h, S2 and S4A–E, and Table S4). The postmitotic neuronal population contained glutamatergic, GABAergic and dopaminergic neurons (Figs. 1h and S4A–C). Among the progenitor population, cells were classified into three clusters consisting of dorsal progenitors, ventral progenitors, and midbrain progenitors + neuroectodermal cells (Figs. 1h and S4D, E). The proportions of glutamatergic or dopaminergic neurons among postmitotic cells were markedly lower in DT1_A than in DT1_U. In contrast, DT1_A organoids contained significantly more GABAergic neurons than DT1_U organoids (Figs. 1i, j and S4F, G). Although there was no significant difference in the dorsal progenitor proportion, DT1_A organoids included notably more ventral progenitors than DT1_U organoids. Alternatively, remarkably fewer midbrain progenitor + neuroectodermal cells were found in DT1_A organoids than in DT1_U organoids (Figs. 1i, k and S4F, H).

We next analyzed differentially expressed genes (DEGs) between the twins (Table S8). Compared with the gene expression in DT1_U organoids, 350 genes were downregulated, and 219 genes were upregulated in DT1_A organoids. IPA of those DEGs revealed significant downregulation of cell cycle controlling pathways and Wnt signaling pathways in DT1_A. Conversely, “GABA Receptor Signaling” was clearly enriched among the upregulated genes in the affected twin. Moreover, there was a significant upregulation of genes regulating axon guidance in DT1_A organoids (Table S9).

### Activation of the Wnt signaling pathway restores the deficit in cell proliferation and the excess GABAergic specification in the affected twin-derived cerebral organoids

**Fig. 2:**
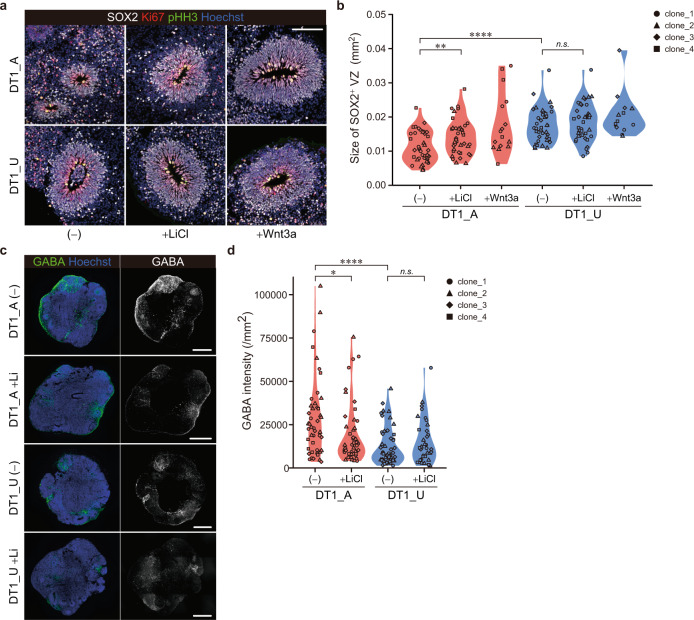
Activation of the Wnt signaling pathway restores the deficit in the size of the VZ-like layer structure and the excess GABAergic specification in the affected twin-derived cerebral organoids. **a** Sample images of immunostaining of organoids from DT1_A and DT1_U on day 30. Scale bar, 100 μm. **b** Quantification of the size of the SOX2^+^ VZ-like layer. Wnt3a-treated samples were not subjected to statistical analyses due to the limited sample size. **c** Sample images of immunostaining of DT1_A and DT1_U organoids showing the GABAergic population on day 30. Scale bars, 500 μm. **d** Quantification of the fluorescence intensity of the GABAergic population. Two-way ANOVA with post hoc *t*-test: ****p < 0.0001; **p < 0.01; *p < 0.05; *n.s.* not significant (**b**, **d**). See also Figs. S5 and S6.

To confirm the difference in the cellular composition during corticogenesis between the twins, we performed immunocytochemical analyses of the organoids. On day 30, cerebral organoids displayed a layered structure consisting of a SOX2^+^ VZ-like layer representing proliferative neural NPCs and a DCX^+^ neuronal layer (Fig. S5A, B). Consistent with the reduced proliferative progenitor population identified in scRNA-seq analysis, DT1_A organoids exhibited significantly smaller SOX2^+^ structures than DT1_U-derived organoids (Figs. 2a, b and S5C–E). In addition, we confirmed the increased GABAergic population in DT1_A organoids compared with that in DT1_U organoids (Figs. 2c, d and S6A, B). To explore the contribution of the diminished Wnt signaling pathway to these events, we activated the pathway by treating the cells with LiCl, a GSK3β inhibitor, or Wnt3a. Interestingly, activation of Wnt/β-catenin signaling during the early neurodevelopmental stage clearly restored both the size of the SOX2^+^ layer and GABAergic neuronal proportion, especially in DT1_A organoids (Figs. 2a–d, S5D, E and S6A, B), suggesting that the decreased Wnt/β-catenin signaling activity affects the cell proliferation and fate specification in the developing brain of a psychotic patient. We confirmed the effect of variability among each iPSC clone on these findings was smaller than the effects of disease status or LiCl treatment (Figs. S5E and S6B and Table S6).

### Diminished Wnt signaling precedes the reduced NPC proliferation and the altered cell fate specification during early neurodevelopmental stages in the affected twin

**Fig. 3:**
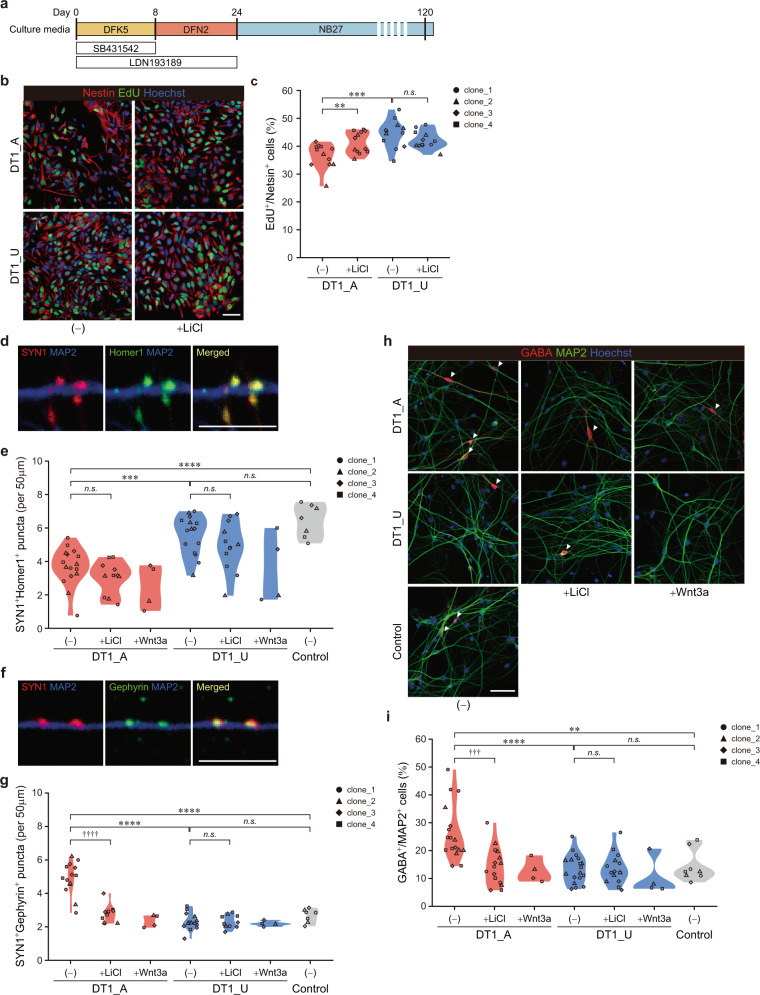
Affected twin-derived neuronal cells exhibit reduced proliferation at the early stage of neurodevelopment and altered excitatory/inhibitory balance in differentiated neural networks at later stages. **a** Schematic protocol for the differentiation of iPSCs into forebrain-specific neuronal cells. **b** Representative images of EdU and Nestin staining in 30-day-old NPCs. Scale bar, 50 μm. **c** Quantitative analysis of EdU-positive proliferating NPCs. Two-way ANOVA with post hoc *t*-test: **p < 0.01; ***p < 0.001; *n.s.* not significant. **d** Representative images of excitatory synapses expressing SYN1 and Homer1 on MAP2^+^ dendrite of 120-day-old neurons. Scale bar, 10 μm. **e** Quantification of SYN1^+^Homer1^+^ excitatory synapses. **f** Representative images of inhibitory synapses expressing SYN1 and Gephyrin on MAP2^+^ dendrite of 120-day-old neurons. Scale bar, 10 μm. **g** Quantification of SYN1^+^Gephyrin^+^ inhibitory synapses. **h** Sample images of immunostaining for GABA and MAP2 from 120-day-old neurons. Scale bar, 50 μm. Arrowheads indicate GABAergic neurons. **i** The proportion of GABA^+^MAP2^+^ cells. One-way ANOVA with Tukey’s multiple comparison test: **p < 0.01; ***p < 0.001; ****p < 0.0001; *n.s.* not significant; two-way ANOVA with post hoc *t*-test: ^†††^*p* < 0.001; ^††††^*p* < 0.0001; *n.s.* not significant; Wnt3a-treated samples were not subjected to statistical analyses due to the limited sample size. See also Fig. S8.

We next identified the order of cellular and molecular alterations observed in the affected twin-derived organoids by time-point profiling with monolayer neuronal cells. The iPSCs of the twins were differentiated into forebrain neural stem/progenitor cells (NSPCs) via the SFEBq method combined with dual SMAD inhibition (Fig. 3a) [32]. We first performed bulk RNA-seq of iPSC-derived NSPCs from two different developmental stages. On day 8, between DT1_A and DT1_U, 150 genes were differentially expressed with our criteria (|fold change| > 1.5 and false discovery rate <0.05), and pathway analyses showed significant enrichment of those DEGs in “Wnt/β-Catenin Signaling” (Table S10). Among the DEGs in this pathway, inhibitors of the signaling pathway [39–41] were upregulated, and a Wnt receptor and its downstream genes were downregulated in DT1_A-derived NSPCs (Fig. S7A), implying diminished Wnt signaling activity during early neurodevelopment in a psychotic patient. We investigated the proliferation and viability of NSPCs at this time point and found no differences between the twins (Fig. S7B, C). On day 24, we found no DEGs meeting the criteria between the twins. However, when we investigated the expression pattern of genes regulating GABAergic neuron specification, key transcription factors *DLX1* and *DLX2* [42] were significantly upregulated in DT1_A-derived NPCs compared with those in DT1_U-derived cells, which was confirmed by RT-qPCR (Fig. S7D, E). In contrast, there was no difference in the expression of *PAX6* and *NEUROG2*, genes regulating the generation of cortical excitatory neurons [43], between the twins (Fig. S7F, G). The expression of neither *DLX1* nor *DLX2* differed between the twins on day 8 (data not shown). Furthermore, the most genes included in the “GABA Receptor Signaling” pathway on the scRNA-seq analysis of the organoids already showed significant upregulation in 24-day-old NPCs from DT1_A (Fig. S7H). In addition, activation of Wnt/β-catenin signaling during early neurodevelopment by treatment with LiCl or Wnt3a tended to suppress the expression of GABAergic specification-associated genes specifically in DT1_A-derived NSPCs (Fig. S7D, E and H). Thus, in the affected twin, Wnt signaling activity was diminished during the very early developmental stage and caused the unbalanced differentiation of GABAergic neurons in cortical development.

We then evaluated the proliferation of forebrain NPCs on day 30. Although there was no difference between the twins on day 8 (Fig. S7C), consistent with the findings from cerebral organoids (Figs. 1e, f and 2a, b), DT1_A-derived NPCs were remarkably less proliferative than those derived from DT1_U at this later stage. LiCl had no obvious effect on DT1_U NPCs, whereas it significantly improved cell proliferation in DT1_A NPCs (Figs. 3b, c and S7I, J), confirming that decreased Wnt signaling activity affected the proliferation of neuronal cells during cortical development in DT1_A. We confirmed the iPSC’s clonal variation effect on NPC proliferation was smaller than the effects of disease status or LiCl treatment (Fig. S7J and Table S6).

### Affected twin-derived neuronal networks exhibit altered excitatory/inhibitory balance at later neurodevelopmental stage

To examine the consequences of the upregulation of genes regulating GABAergic neuron differentiation observed in DT1_A-derived NPCs during early developmental stages, we further differentiated the cells into mature cortical neurons exhibiting spontaneous firing (Fig. S8A). Whereas soma size and neuronal dendritic arborization did not differ between 120-day-old neurons from the twins (data not shown), DT1_A neurons exhibited more dendritic protrusions than DT1_U neurons (Fig. S8B), suggesting an accelerated maturation of the patient’s neurons. We then investigated the balance of neuronal subtypes among iPSC-derived cortical networks by electrophysiological and immunocytochemical analyses on day 120. The neurons of the twins did not show any notable differences in the frequency or amplitude of excitatory or inhibitory synaptic currents (Fig. S8C–H). However, immunolabeling neurons with the presynaptic marker synapsin-1 (SYN1) and the postsynaptic markers Homer1 (for excitatory synapses) or Gephyrin (for inhibitory synapses) revealed an imbalance of excitatory and inhibitory synaptic density between the twins. DT1_A neurons exhibited a marked decrease in the density of SYN1^+^Homer1^+^ excitatory synapses (Figs. 3d, e and S8I,J). Conversely, there was a significant increase in SYN1^+^Gephyrin^+^ inhibitory puncta in DT1_A compared with DT1_U-derived neurons (Figs. 3f, g and S8K, L). Moreover, the proportion of GABAergic neurons was markedly higher in DT1_A than in DT1_U (Figs. 3h, i and S8M,N). Activation of Wnt signaling by LiCl treatment during the early stage (days 4–24) exerted no obvious effects on the density of excitatory synapses in either twin (Figs. 3e and S8I). However, specifically in DT1_A neurons, LiCl significantly reduced the density of inhibitory synapses and the GABAergic population (Figs. 3g–i and S8K,M). Importantly, cortical neurons derived from iPSCs of healthy control individuals (Figs. S1A and S8O) behaved indistinguishably from DT1_U neurons, whereas they exhibited significant differences in both synaptic densities and GABAergic proportion from DT1_A neurons (Figs. 3e, g, i and S8I, K, M). This finding suggests that the increased GABAergic specification phenotype is indeed associated with psychosis. Thus, diminished Wnt/β-catenin signaling during the early stages of neurodevelopment leads to a neurodevelopmental shift toward increased inhibition in the excitation and inhibition (E/I) balance of in vitro networks formed from DT1_A-derived cortical neurons at the later stage. We confirmed the effect of variability among each iPSC clone on these phenotypes was smaller than the effects of disease status or LiCl treatment (Fig. S8J, L, N and Table S6).

### iPSC-derived NPCs from three monozygotic twins with psychoses show upregulation of GABAergic synapse-related genes compared with their healthy co-twins

**Fig. 4:**
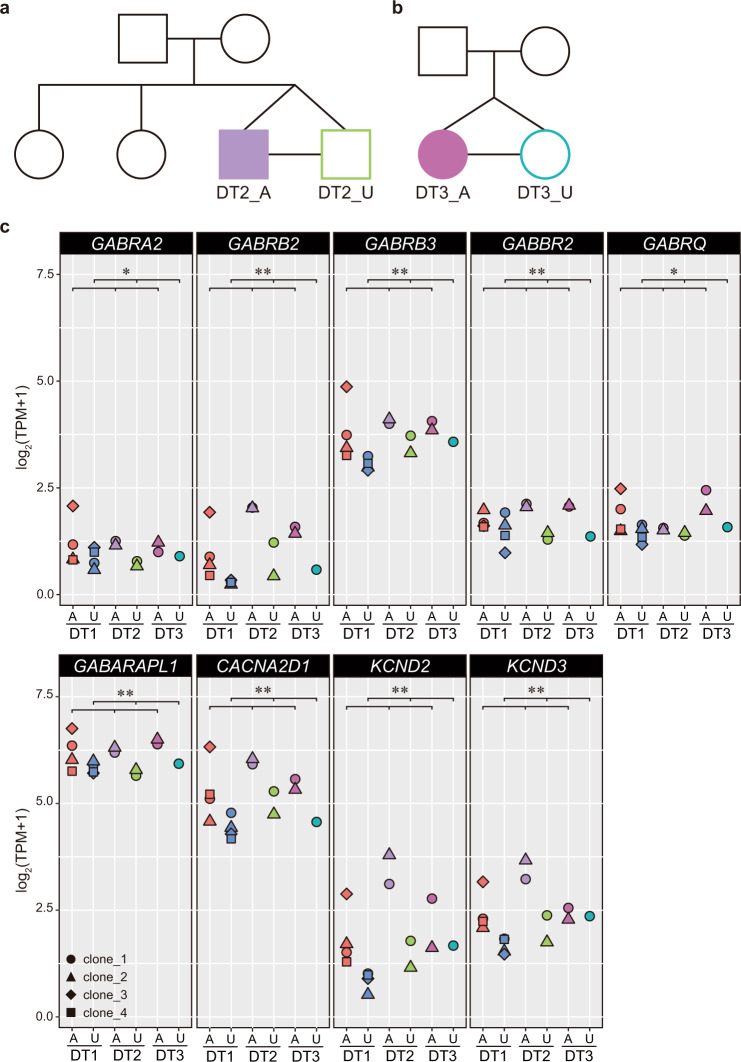
GABAergic synapse-related genes were significantly upregulated in iPSC-derived NPCs from three monozygotic twins with psychoses compared with the expression in their healthy co-twins. **a**, **b** Diagrams of the pedigree including a pair of monozygotic twins discordant for SZ. DT2_A and DT3_A, affected twins; DT2_U and DT3_U, unaffected twins. **c** Gene expression levels of GABAergic synapse-related genes from bulk RNA-seq analysis. Representative genes encoding GABA receptor subunits and ion channels are shown. A affected twin, U unaffected twin. Welch’s *t* test: *p < 0.05; **p < 0.01. See also Fig. S9.

Taken together, these findings suggest that abnormally enhanced GABAergic specification during cortical development is one of the cellular endophenotypes of psychosis. To further evaluate the relevance of this phenotype to the disorder, we analyzed two additional pairs of monozygotic twins discordant for SZ (Figs. 4a, b and S1A). iPSCs were established from LCLs of each individual (Fig. S9A,B) and differentiated into forebrain NPCs (Fig. 3a). We then performed bulk RNA-seq and compared the expression patterns of genes annotated with at least one of the terms “GABAergic Synapse” in KEGG pathway (hsa04727) or Gene Ontology (GO: 0098982) and “GABA Receptor Signaling” in IPA among three patients with psychoses and their healthy co-twins (DT1, DT2, and DT3) on day 24. Among 146 genes analyzed, 37 genes were significantly differentially expressed between the affected twins and the unaffected twins (Table S11). Compared with the NPCs from the unaffected twins, the NPCs from all three twins affected by psychoses exhibited upregulation of 35 genes, including genes encoding both pre- and postsynaptic GABA receptor subunit and ion channels (Fig. 4c), and downregulation of only two genes (Fig. S9C and Table S11). Thus, there was a significant upregulation of genes associated with GABAergic synapses during early neurodevelopment between the twins affected with psychoses and their co-twins (*p* < 1.0244e-08, Fisher’s exact test).

## Discussion

Although decades of research have suggested several neurodevelopmental mechanisms underlying SZ and BD, substantial genetic heterogeneity among individuals has hampered the identification of specific disease-associated cellular phenotypes and their temporal order in the pathogenesis of these disorders, especially in sporadic cases that represent the vast majority of the patients. Therefore, analyzing patient neurons by simulating brain development with a well-controlled genetic background has been required.

In this study, with cerebral organoids and forebrain NPCs derived from iPSCs of monozygotic twins discordant for psychoses, we propose the disease-associated staged dysregulation of neuronal development in which deficits in Wnt signaling pathway precede the reduced proliferation and marked dorsal-ventral fate shift to neuronal network E/I balance tilted toward GABAergic interneuron differentiation. Previous studies have reported the alterations in neuronal fate specification toward increased ventralization/GABAergic differentiation in iPSC-derived neuronal cells from the patients with BD [44, 45]. Chen et al. [44] demonstrated the enhanced ventralization during early neurodevelopment of the patient-derived cells, while the neurons of healthy individuals were more shifted to the dorsal fate. They also showed that LiCl can promote dorsalization of the patients’ neurons. This finding was confirmed by Kim et al. [45] in which patient-derived neurons exhibited higher expression of *GAD1* and increased GABAergic specification compared with healthy individuals. Even though these studies including ours utilized different neuronal differentiation protocols and analyzed completely independent patient cohorts, each study observed the similar phenotype of enhanced GABAergic specification in iPSC-derived neurons form patients with BD, implying that the alterations in neuronal fate specification is one of the mechanisms underlying the disorder. In 24-day-old NPCs derived from DT1_A, gene cohorts important for the differentiation of GABAergic interneurons were upregulated (Fig. S7D, E,H) in accordance with our functional studies indicating increased numbers of GABAergic neurons and inhibitory synapses in neural networks in vitro (Figs. 2d and 3g, i). During early neural development, Shh and Wnts act antagonistically in the dorsal-ventral patterning of the neural tube [46]. Interestingly, our RNA-seq analyses of iPSC-derived NSPCs identified that *SHH* tended to be upregulated in DT1_A on day 8 (log2FC = −0.77339, *Padj* = 0.07), in addition to diminished Wnt signaling (Fig. S7A). These alterations suggest that dysregulated Wnt/β-catenin signaling enhanced the ventralization of the affected twin’s neuronal cells via Shh signaling.

In the present study, while DT1_A-derived neurons exhibited significant increase in the inhibitory synaptic puncta and marked decrease in the excitatory puncta compared with DT1_U neurons (Fig. 3e, g), they did not show any obvious electrophysiological abnormalities except for the significant reduction of mIPSC amplitude by LiCl in DT1_A-derived neurons (Fig. S8D, E, G, H). One possible mechanism underlying this contradiction is the functional maturity of iPSC-derived neurons. Since protein and gene expression might precede electrophysiological properties by days or even weeks, it is possible that iPSC-derived neurons in our study is not functionally fully matured even they expressed synaptic markers. Indeed, DT1_A neurons had remarkably more Gephyrin puncta than DT1_U (Fig. S10A) suggesting an increased GABAergic release on dendrites in DT1_A-derived neuronal networks [47] which supports our finding of the increase in GABAergic population in DT1_A neurons. On the other hand, consistent with our findings on the mIPSC amplitude, the size of Gephyrin puncta did not differ between the twins (Fig. S10B), suggesting that there is no obvious difference between the twins in the functional/electrophysiological maturity of synapses on iPSC-derived neurons. LiCl significantly decreased both the density and size of Gephyrin puncta especially in DT1_A neurons (Fig. S10A, B). The significant attenuation of mIPSC amplitude in LiCl-treated DT1_A neurons might reflect both reduction and immaturation of the inhibitory synapses by LiCl.

Previous studies analyzing iPSC-derived neurons from the patients with SZ have suggested the alterations in synaptic function such as attenuation of neuronal connectivity [48], reduced neuronal activity [49–51], deficit in synaptic density [48, 49, 51–53], and neurotransmitter release [49, 52, 54]. However, these findings relied on the analyses of spontaneous synaptic events and morphology. Although spontaneous events are insufficient predictor of synaptic maturity and function, there are few reports tested evoked synaptic transmission with neurons classically (i.e., not transcription factor-based) differentiated from human PSCs, suggesting the limitation of studying mature neuronal properties, such as synaptic transmission, with those PSC-derived neurons. Classically differentiated PSC-derived neurons as analyzed in the present study have heterogeneity of maturation states [55] and it might cause the difficulty in investigating synaptic transmission. On the other hand, transcription factor-based induced neuronal cells (iNs) are reported to exhibit higher degree of synaptic maturation than classical PSC-derived neurons and evoked EPSC and IPSC [56, 57]. To date, by analyzing the evoked synaptic events, several studies have demonstrated failures in neurotransmitter release in iNs from PSCs carrying disease-associated gene mutation such as *STXBP1* which causes infantile early epileptic encephalopathy [58] and *NRXN1* associated with autism and SZ [59]. Moreover, recent studies developed an autaptic culture system for human iPSC-derived neurons for the analysis of evoked synaptic transmission and short-term plasticity [60, 61]. Thus, further investigation of the evoked synaptic events on twins’ iPSC-derived neurons with those alternative system might support or strengthen our findings of the altered E/I balance of in vitro networks in DT1_A neurons.

Several studies of postmortem brains from patients with SZ, schizoaffective disorder, and/or BD have demonstrated a preferential decrease in the number of interneurons in both the cerebral cortex and hippocampus, suggesting that these disorders are associated with disruptions in GABAergic networks [62]. Our analysis of postmortem brain tissues from patients with psychoses and non-psychiatric control individuals (Fig. S11A) [35] also demonstrated significant downregulation of GABAergic synapse-related genes in the patient brains. Among the 146 genes investigated, 107 genes exhibited decreased expression, and only 11 genes were upregulated in the psychotic brains (*p* < 6.4038e-21, Fisher’s exact test) (Fig. S11B and Table S11). The enhanced GABAergic specification observed in iPSC-derived neuronal cells from the patient with psychoses (Figs. 4c and S9C) is seemingly inconsistent with those findings. However, postmortem brain data are an end stage of the disease and reflect consequences of a life-long illness and pharmacological intervention. Thus, the specific perturbations during early brain development that underlie the reduction in GABAergic neurons in adults remain unclear. Human iPSC-derived neuronal cells recapitulate cells of the fetal brain [27, 63]; therefore, iPSC-based models of neuronal disorders provide us an opportunity to investigate molecular and cellular alterations before disease onset. Moreover, the pathological processes likely differ between the early developmental stage and adulthood. For example, in autism, brain overgrowth has been reported in early development, but this is followed by premature arrest of growth, resulting in smaller brain volume in adults [64]. Thus, an excess of GABAergic neurons during early development may be followed by marked decreases in the GABAergic neuronal population in adults after disease onset. Furthermore, diminished Wnt signaling activity during the very early neurodevelopmental stage might affect the brain volume of DT1_A before the onset. Studies of individuals at high risk for SZ consistently showed that cerebral cortex gray matter volume is smaller in this population compared with controls [65], suggesting that brain volume is already small at the onset of illness, and further reduction of gray matter volume occurs after the onset. However, the prodromal studies of SZ also suggested that brain shrinkage mainly takes place in the first 1.5 years after the onset [66]. Thus, the correlation between the cellular phenotype and the changes in morphology of patient’s brain remains controversial.

Studies using iPSC-derived neuronal cells from patients with SZ or BD have reported changes in Wnt signaling pathways [45, 48, 67, 68] altered neural fate specification [44, 45], mitochondrial abnormalities [69], dysregulated neuronal activity [49–51] and excitability [69, 70], and synaptic dysfunctions [48, 49, 51–54]. A recent study using cerebral organoids also showed altered excitability of BD patients-derived neurons [71]. However, the relationships among the alterations and their order have not been well investigated. Our scRNA-seq analysis of cerebral organoids and additional functional genomic analyses with time-point profiling of iPSC-derived neuronal cells enabled concluding that pathogenic decreases in cell proliferation and enhanced GABAergic specification temporally follow defective Wnt/β-catenin signaling pathway in iPSC-derived neuronal cells from a patient with SA-B. Premature commitment to neuronal differentiation resulting from reduced progenitor proliferation and excess network inhibition during cortical development may cause an E/I imbalance in later adult neuronal network function.

In the current study, the fundamental cause of discordance in the cellular phenotypes between the twins remain unclear. To identify a genomic discordance between DT1_A and DT1_U, we performed whole genome sequencing analysis of peripheral blood samples and have not identified any obvious genomic differences, such as single nucleotide variants, insertions and deletions, and long interspersed element-1 (LINE-1) insertion. On the other hand, based on DNA methylation analyses, we identified a CpG site which were differentially methylated throughout blood to iPSC-derived NPCs between the twins. It might suggest a contribution of epigenetic differences which are maintained throughout the reprogramming process to the discordance in cellular phenotypes in DT_1 pair. Further investigations including whole genome DNA methylation analysis might resolve this complexity.

Based on a strategy to minimize genetic variability by analyzing monozygotic twin pairs discordant for the disorders, the current study proposes that a shift in the balance of neuronal and synaptic prevalence toward GABAergic specification during early cortical development is one of the endophenotypes of psychoses. Further studies to clarify the correlation between the cellular phenotype and the patient’s symptoms is an essential issue to be addressed for understanding of the disease pathogenesis and discovering novel therapeutic targets. Moreover, future studies utilizing the methodology used in this study will focus on reconstituting the entire disease cascade for psychoses, while the parallel accumulation of data from iPSC lines of other patients may lead to an understanding of the general principles underlying complex, variegated psychiatric disorders.

## Supplementary information

Supplementary Info

Supplementary Tables

## Data Availability

All datasets generated during the current study are available from the Lead Contact upon reasonable request. Single-cell RNA sequencing data that support the findings of this study have been deposited at the Gene Expression Omnibus (GEO) under accession number GSE120190.

## Code availability

All codes generated during the current study are available from the Lead Contact upon reasonable request.
