# Supplementary material for: Developmental excitation-inhibition imbalance underlying psychoses revealed by single-cell analyses of discordant twins-derived cerebral organoids
Source: Mol Psychiatry. 2020 Aug 7;25(11):2695–711. doi: 10.1038/s41380-020-0844-z (PMC7577852; doi:10.1038/s41380-020-0844-z)
Supplement: Supplementary file 1 — Supplementary Info [file 41380_2020_844_MOESM1_ESM.pdf]

Supplementary Information for

Developmental Excitation-Inhibition Imbalance Underlying Psychoses Revealed by Single-Cell Analyses of Discordant Twins-Derived Cerebral Organoids

Tomoyo Sawada*, Thomas E. Chater, Yohei Sasagawa, Mika Yoshimura, Noriko Fujimori-Tonou, Kaori Tanaka, Kynon J. M. Benjamin, Apuã C. M. Paquola, Jennifer A. Erwin, Yukiko Goda, Itoshi Nikaido, Tadafumi Kato*

*Correspondence to: Tomoyo.Sawada@libd.org, tadafumi.kato@juntendo.ac.jp

This PDF file includes:

Figures S1 to S11

Supplementary References

Another Supplementary Material for this manuscript includes the following:

Tables S1 to S11 (Excel file)

A

Subjects	Diagnosis (Dx)	Dx criteria	Age	Onset	Sex	Affected Twin	Unaffected Twin	Parental cells for iPSC	Family History	Note
DT1	Schizoaffective disorder, bipolar-type	DSM-IV, MINI	20's	20's	M	DT1_A	DT1_U	PBMCs	None	
DT2	Schizophrenia	DSM-IV	50's	20's	M	DT2_A	DT2_U	LCLs	None	
DT3	Schizophrenia	DSM-IV	20's	10's	F	DT3_A	DT3_U	LCLs	None	
C1	No mental disorder	MINI	50's	n/a	F			PBMCs	None (except for DT1_A)	DT1's mother
C2	No mental disorder	MINI	50's	n/a	M			PBMCs	None (except for DT1_A)	DT1's father
C3	No mental disorder	MINI	50's	n/a	M			PBMCs	None	unrelated individual
C4	No mental disorder	MINI	10's	n/a	M			PBMCs	None	unrelated individual

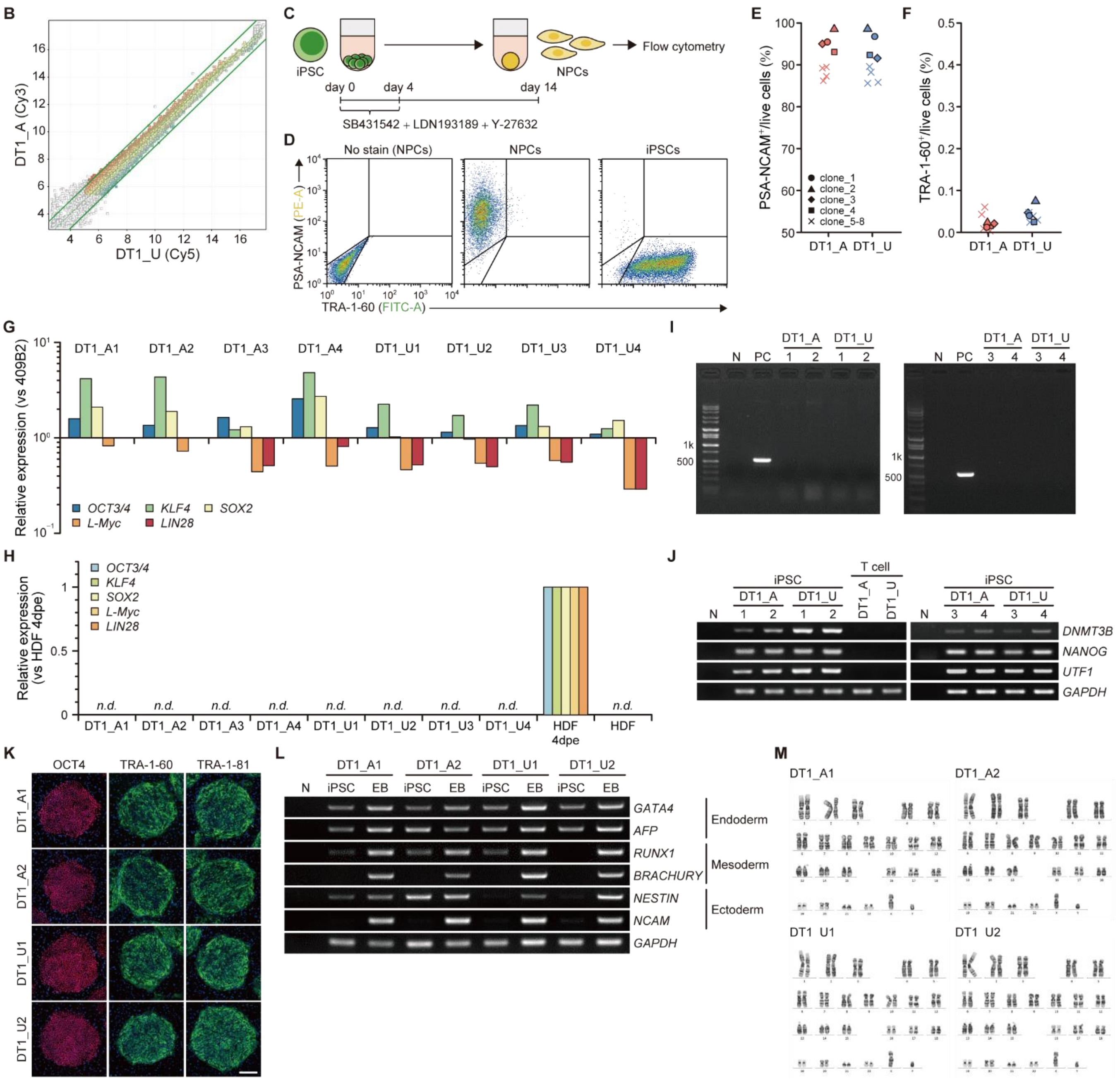

Figure S1| Characterization of iPSCs derived from DT1

(A) List of the participants in this study and their clinical information. DSM-IV, Diagnostic and Statistical Manual of Mental Disorders; MINI, Mini-International Neuropsychiatric Interview; n/a, not applicable.

(B) Scatter plot of array CGH analysis of genomic DNA from peripheral blood of DT1_A and DT1_U. Green lines correspond to fold change = 2; white squares indicate signals flagged ‘Undetected’ or ‘Compromised’. No candidate genomic regions for copy number difference were identified.

(C) Schematic of the neural induction method used for evaluation of neural differentiation potency.

(D) Representative FACS plots. Almost all the undifferentiated iPSCs were positive for TRA-1-60 (right), whereas the cells two weeks after neural induction became positive for PSA-NCAM and negative for TRA-1-60 (center).

(E and F) The proportion of PSA-NCAM$^+$ cells (E) and TRA-1-60$^+$ undifferentiated cells (F) two weeks after neural induction. Each data represents the average of two independent assays.

(G) Quantification of the expression of pluripotent stem cell marker used for reprogramming in each iPSC clone by RT-qPCR. Total (both of exogenous and endogenous) expression was analyzed. Relative expression levels compared to 409B2 line (set as ‘1’) are indicated.

(H) RT-qPCR results showing that all four iPSC clones from discordant twins do not retain the exogenous reprogramming factors. *n.d.*, not detected; Relative expression levels to HDF 4dpe (human dermal fibroblast, four days post electroporation) are shown.

(I) Genomic PCR results showing no genomic integration of episomal vectors used for reprogramming in twins-derived iPSCs. N, no template; PC, positive control (pCXLE-hSK).

(J) RT-PCR results showing the expression of pluripotent stem cell markers in iPSCs. Parental T cells were used as negative controls; N, no template.

(K) Images of immunostaining of iPSC colonies with pluripotent stem cell markers. Scale bar, 200 μm.

(L) RT-PCR results showing that twins’-derived iPSC clones have potential to differentiate into three germ layers. N, no template; EB, embryoid body.

(M) Representative karyotyping images of established iPSCs showing normal chromosomal structure.

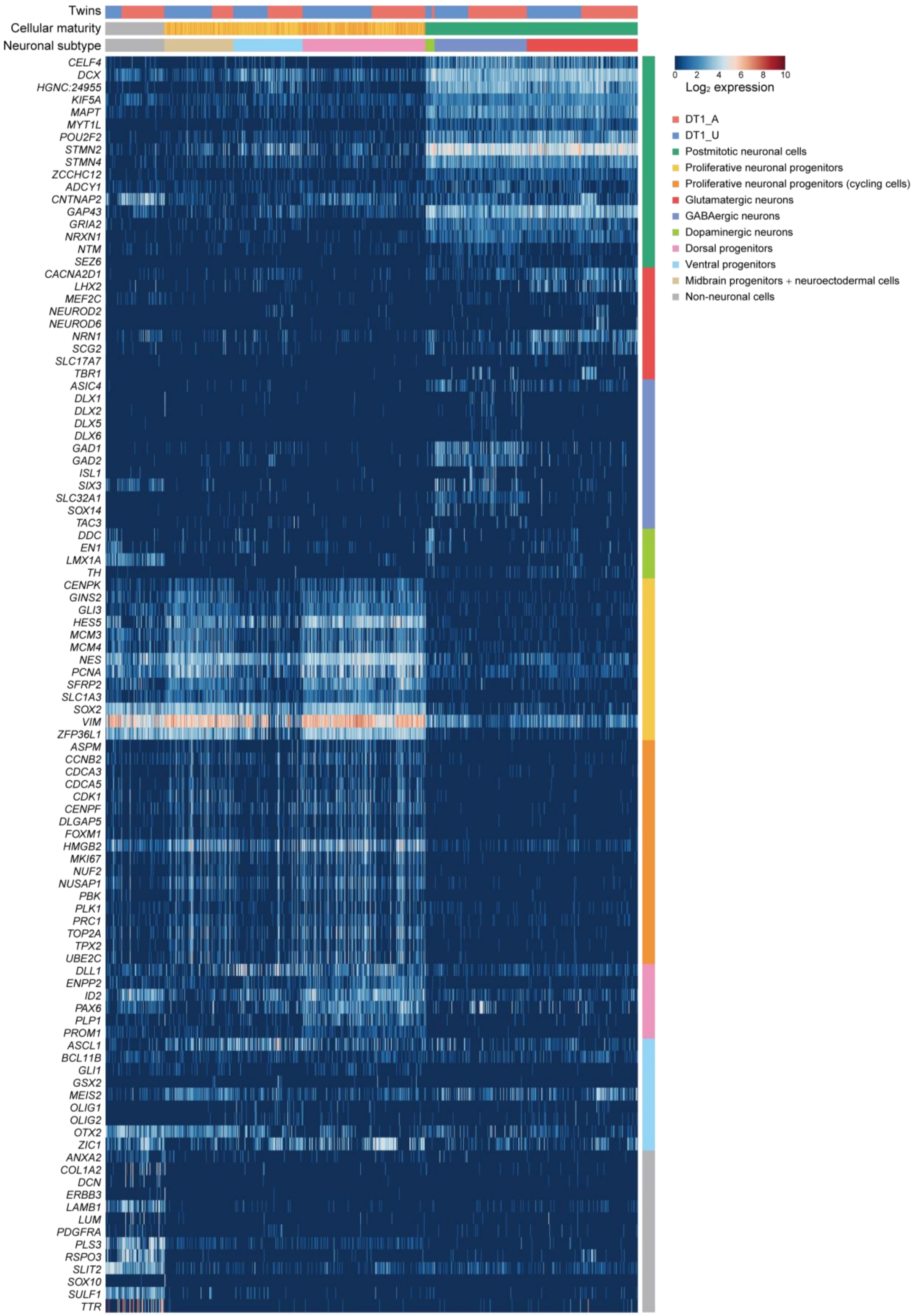

Twins
Cellular maturity
Neuronal subtype
CELF4
DCX
HGNC:24955
KIF5A
MAPT
MYT1L
POU2F2
STMN2
STMN4
ZCCHC12
ADCY1
CNTNAP2
GAP43
GRIA2
NRXN1
NTM
SEZ6
CACNA2D1
LHX2
MEF2C
NEUROD2
NEUROD6
NRN1
SCG2
SLC17A7
TBR1
ASIC4
DLX1
DLX2
DLX5
DLX6
GAD1
GAD2
ISL1
SIX3
SLC32A1
SOX14
TAC3
DDC
EN1
LMX1A
TH
CENPK
GINS2
GLI3
HES5
MCM3
MCM4
NES
PCNA
SFRP2
SLC1A3
SOX2
VIM
ZFP36L1
ASPM
CCNB2
CDCA3
CDCA5
CDK1
CENPF
DLGAP5
FOXM1
HMGB2
MKI67
NUF2
NUSAP1
PBK
PLK1
PRC1
TOP2A
TPX2
UBE2C
DLL1
ENPP2
ID2
PAX6
PLP1
PROM1
ASCL1
BCL11B
GLI1
GSX2
MEIS2
OLIG1
OLIG2
OTX2
ZIC1
ANXA2
COL1A2
DCN
ERBB3
LAMB1
LUM
PDGFRA
PLS3
RSPO3
SLIT2
SOX10
SULF1
TTR
0 2 4 6 8 10
Log2 expression
DT1_A
DT1_U
Postmitotic neuronal cells
Proliferative neuronal progenitors
Proliferative neuronal progenitors (cycling cells)
Glutamatergic neurons
GABAergic neurons
Dopaminergic neurons
Dorsal progenitors
Ventral progenitors
Midbrain progenitors + neuroectodermal cells
Non-neuronal cells

Figure S2| Deconstruction of cellular composition of DT1 iPSC-derived cerebral organoids (related to Figure 1)

Heatmap showing expression of genes that distinguish cellular maturity and regional identity of the different *t*-SNE clusters. The cell type representing the cellular maturity and neuronal cell type that is marked by a given gene set is shown by different color on the left side of the heatmap. Genes are in rows and cells are in columns.

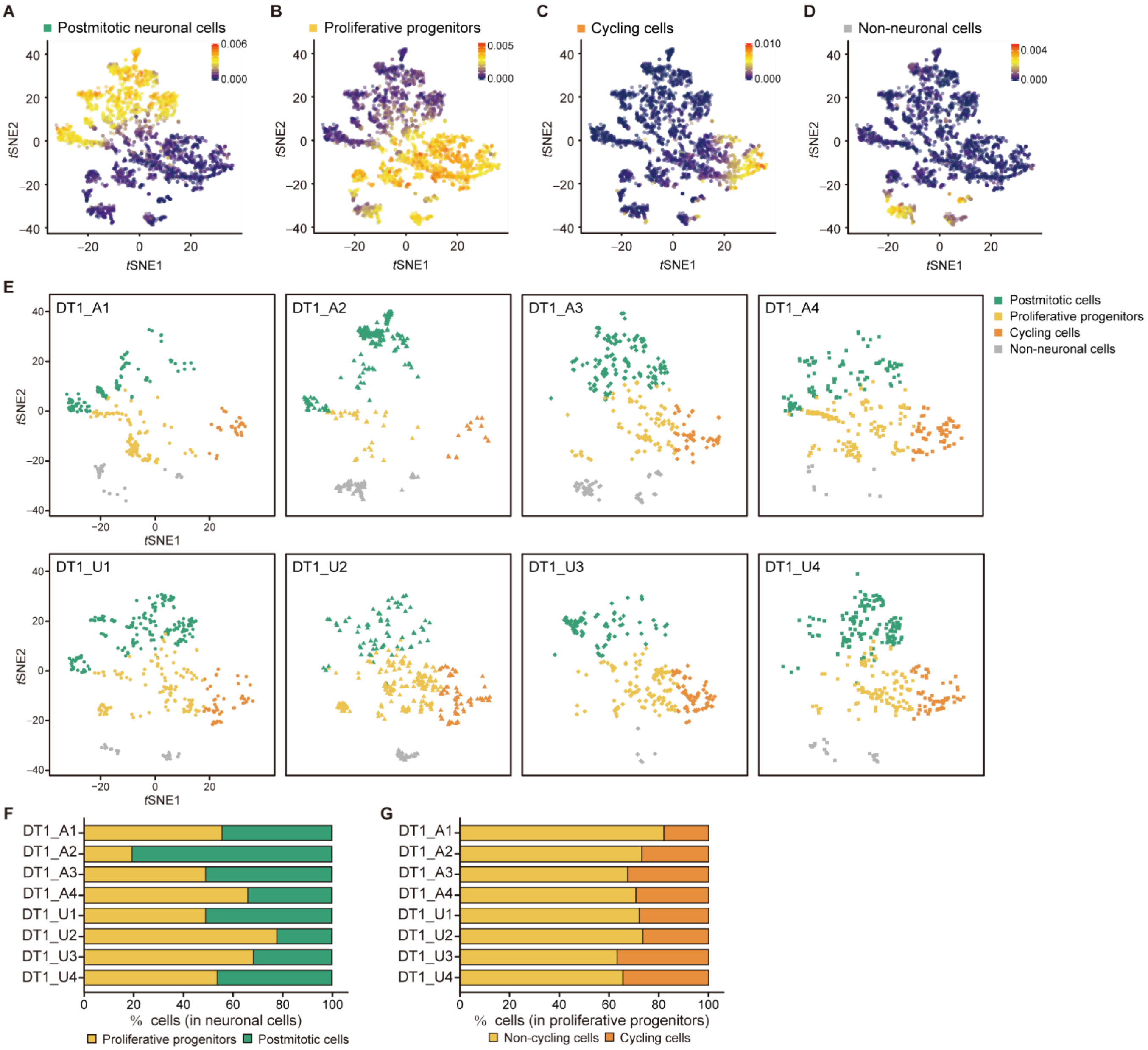

Figure S3| Cellular composition of cerebral organoids based on cellular maturity (related to Figure 1)

(A-D) *t*SNE plots showing the distribution of relative expression of specific marker genes determining the cellular maturity and classifying neuronal and non-neuronal populations. Color intensity indicates the total UMI counts of cell type-specific marker genes/total UMI counts of all genes for each single cell.
(E) *t*SNE plots showing cellular maturity of the organoids derived from each iPSC clone.
(F and G) Proportion of proliferative progenitors and postmitotic cells among the neuronal population (F) and of non-cycling cells and cycling cells among proliferative progenitors (G).

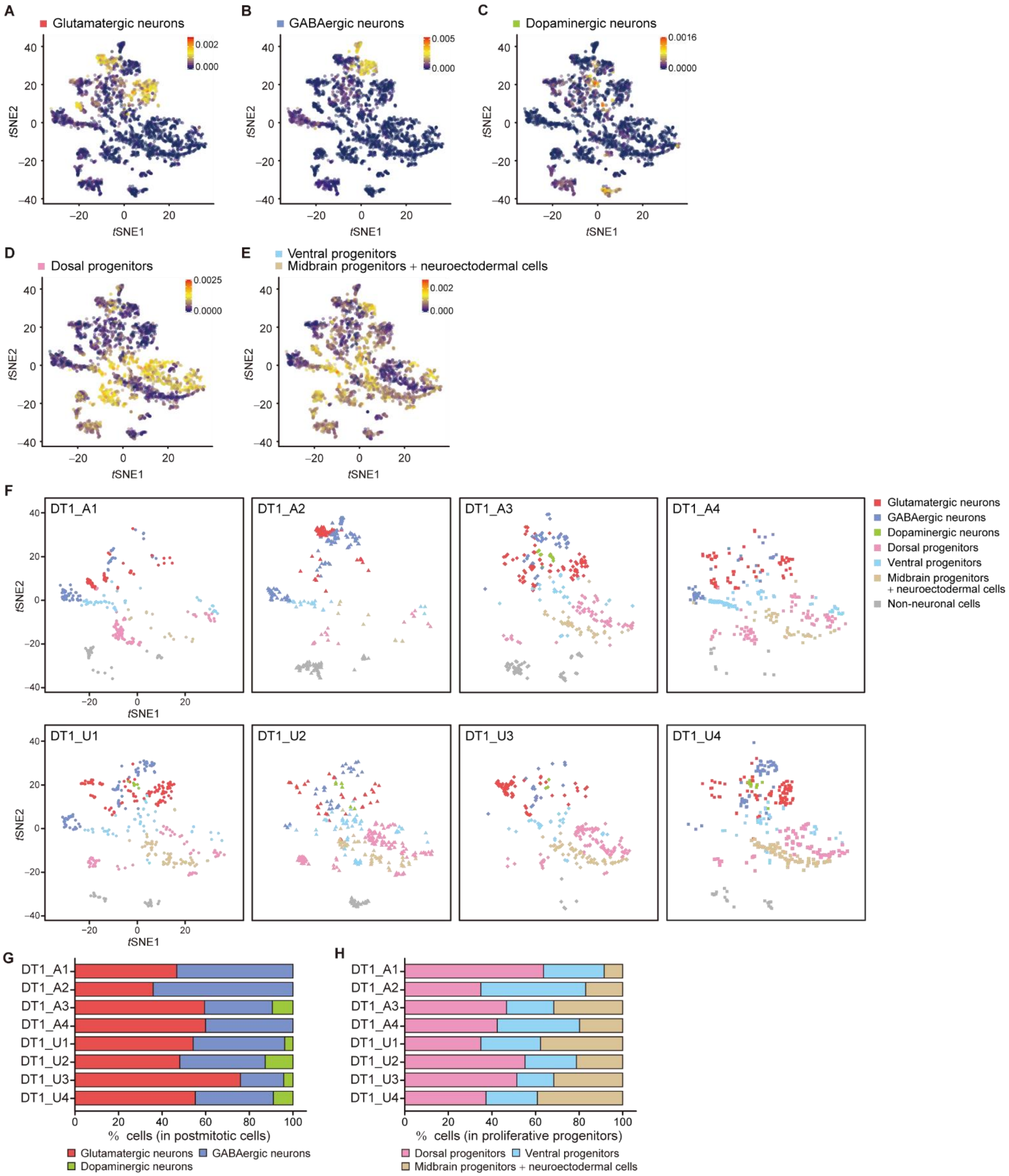
A
Glutamatergic neurons
B
GABAergic neurons
C
Dopaminergic neurons
D
Dosal progenitors
E
Ventral progenitors
Midbrain progenitors + neuroectodermal cells
tSNE1
tSNE2
F
DT1_A1
DT1_A2
DT1_A3
DT1_A4
DT1_U1
DT1_U2
DT1_U3
DT1_U4
Glutamatergic neurons
GABAergic neurons
Dopaminergic neurons
Dorsal progenitors
Ventral progenitors
Midbrain progenitors + neuroectodermal cells
Non-neuronal cells
G
% cells (in postmitotic cells)
Glutamatergic neurons
GABAergic neurons
Dopaminergic neurons
H
% cells (in proliferative progenitors)
Dorsal progenitors
Ventral progenitors
Midbrain progenitors + neuroectodermal cells

Figure S4| Cellular composition of cerebral organoids based on neuronal subtype (related to Figure 1)

(A-E) *t*SNE plots showing the distribution of relative expression of specific marker genes for neuronal subtype. Color intensity indicates the total UMI counts of cell type-specific marker genes / total UMI counts of all genes for each single cell. The cluster of ‘midbrain progenitors + neuroectodermal cells’ are determined based on the expression patterns of *MEIS2* and *OTX2*[27,72,73].

(F) *t*SNE plots showing the distribution of neuronal subtypes of the organoids derived from each iPSC clone.

(G and H) Proportion of neuronal subtypes among the postmitotic population (G) and among the proliferative progenitor population (H).

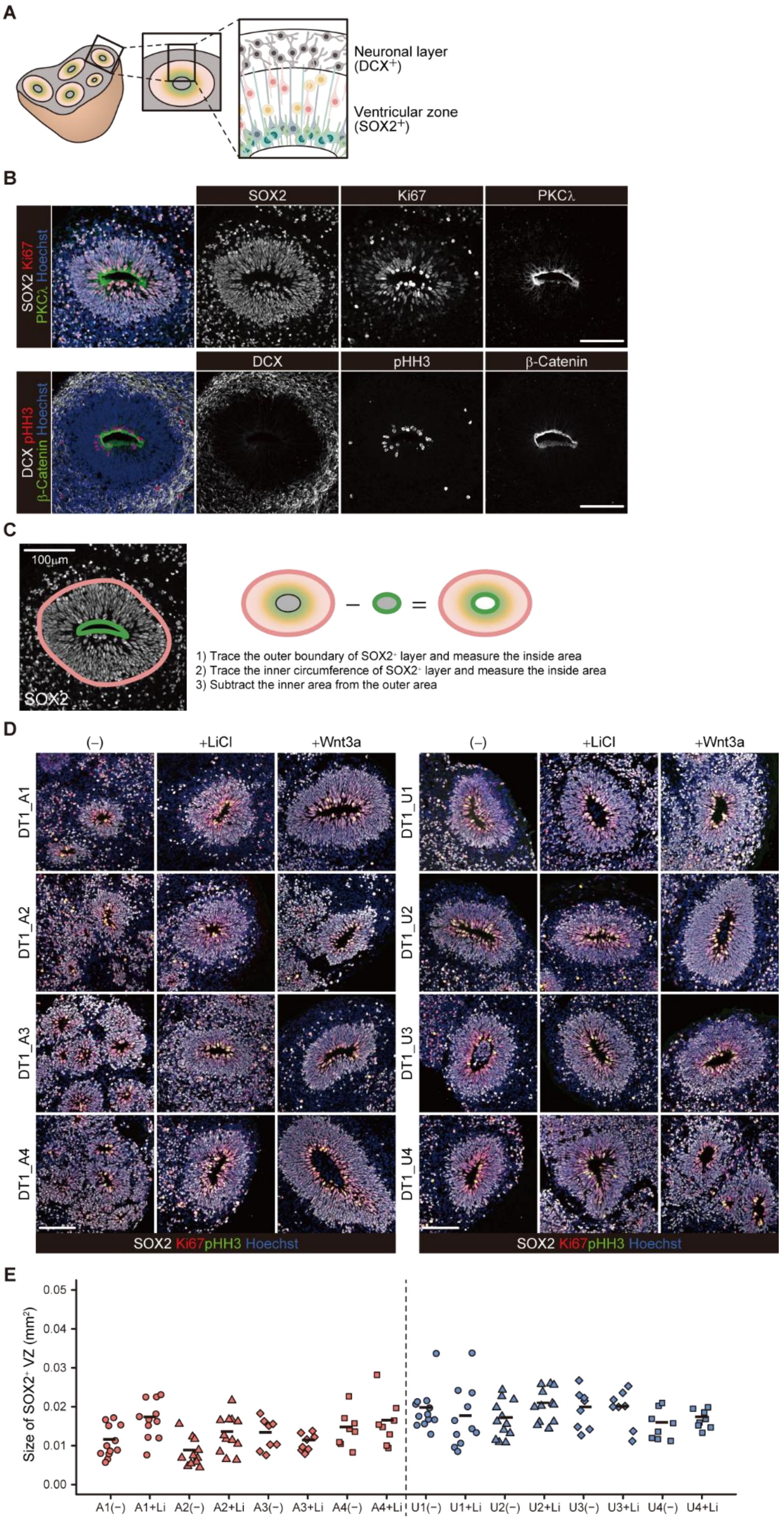
A
Neuronal layer
(DCX+)
Ventricular zone
(SOX2+)
B
SOX2 Ki67 Hoechst
PKCλ
SOX2
Ki67
PKCλ
DCX pHH3 Hoechst
β-Catenin
DCX
pHH3
β-Catenin
C
100μm
SOX2
1) Trace the outer boundary of SOX2+ layer and measure the inside area
2) Trace the inner circumference of SOX2+ layer and measure the inside area
3) Subtract the inner area from the outer area
D
(–)
+LiCl
+Wnt3a
DT1_A1
DT1_A2
DT1_A3
DT1_A4
DT1_U1
DT1_U2
DT1_U3
DT1_U4
SOX2 Ki67 pHH3 Hoechst
E
Size of SOX2+ VZ (mm2)
0.05
0.04
0.03
0.02
0.01
0.00
A1(–) A1+Li A2(–) A2+Li A3(–) A3+Li A4(–) A4+Li U1(–) U1+Li U2(–) U2+Li U3(–) U3+Li U4(–) U4+Li

Figure S5| Morphological analyses of DT1 iPSC-derived cerebral organoids (related to Figure 2)

(A) Schematic representation of organoid structure on day 30.

(B) Sample images of immunostaining of DT1_U organoids on day 30. Within the $SOX2^+$ structure, we observed localization of adherent junction markers (PKCλ and β-Catenin) at the apical surface and proliferating cells expressing Ki67 or phospho-histone H3 (pHH3) around the surface. Scale bars, 500 μm.

(C) Summary of the method for measuring the size of the $SOX2^+$ VZ-like layer.

(D) Sample images of immunostaining of organoids on day 30 related to Figure 2B (images from DT1_A1 and DT1_U3 are represented in Figure 2A). Scale bars, 100 μm.

(E) Quantification of the size of the $SOX2^+$ VZ-like layer related to Figure 2B. Data from each iPSC clone were presented individually. Bars, mean.

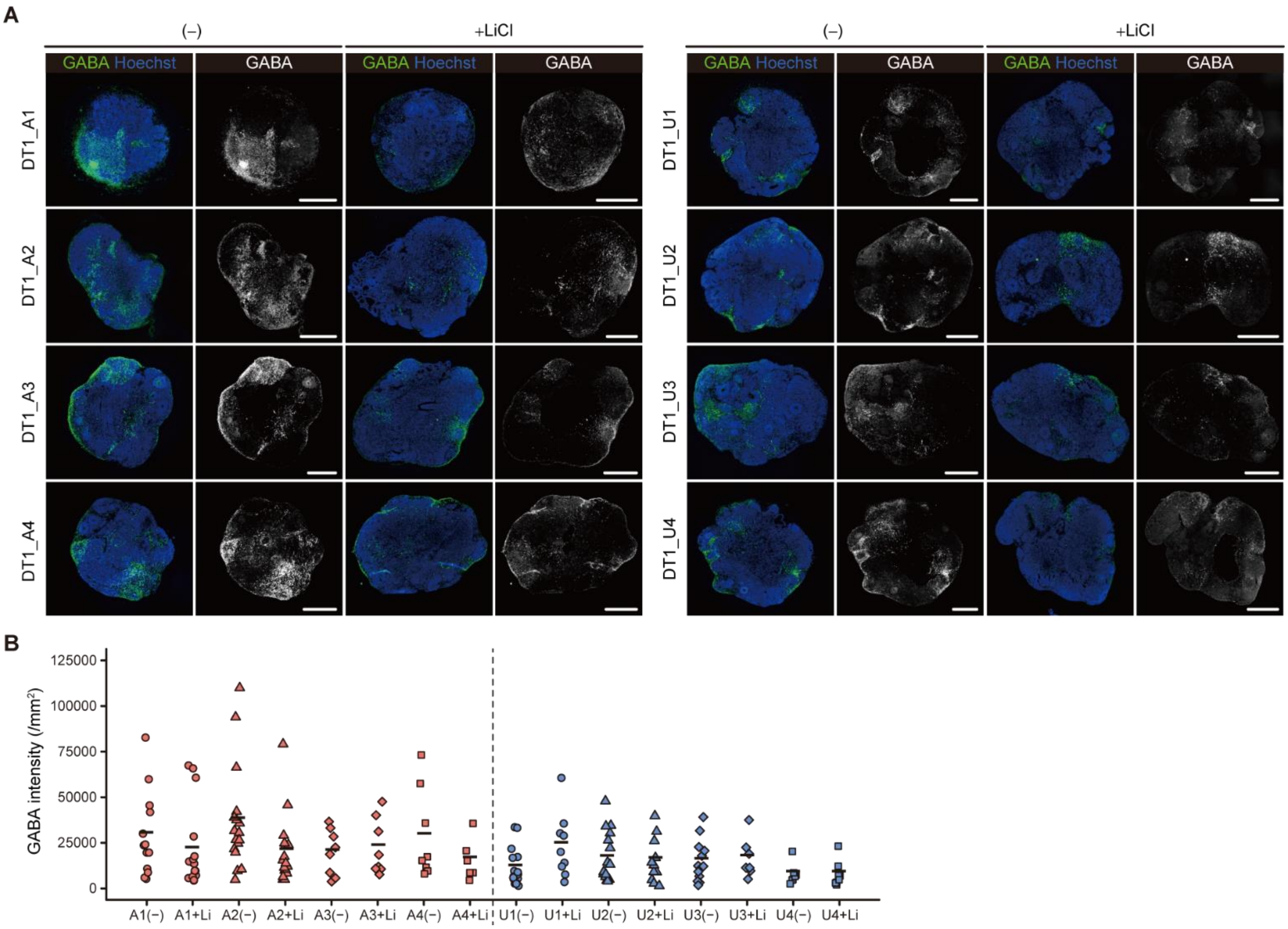

Figure S6| Immunostaining of the cerebral organoids to confirm the excess GABAergic specification in DT1_A (related to Figure 2)

(A) Representative images of immunostaining of organoids on day 30 related to Figure 2D (images from DT1_A3 and DT1_U1 are presented in Figure 2C). Scale bars, 500 μm.

(B) Quantification of the fluorescence intensity of GABAergic population related to Figure 2D. Data from each iPSC clone were presented individually. Bars, mean.

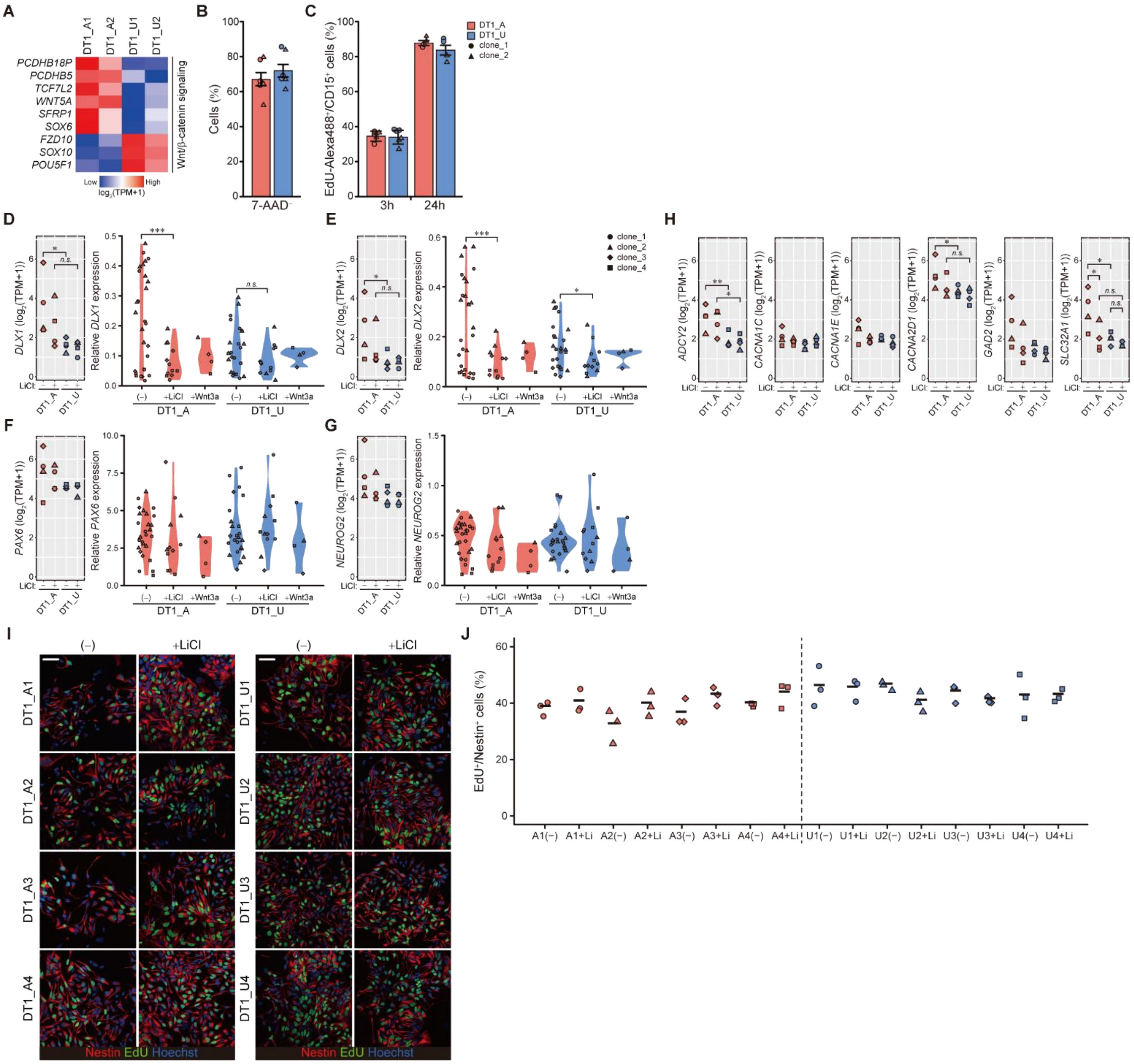

A
DT1_A1
DT1_A2
DT1_U1
DT1_U2
PCDHB18P
PCDHB5
TCF7L2
WNT5A
SFRP1
SOX6
FZD10
SOX10
POU5F1
Wnt/β-catenin signaling
Low
High
log2(TPM+1)
B
Cells (%)
7-AAD-
C
EdU-Alexa488+/CD15+ cells (%)
3h
24h
DT1_A
DT1_U
clone_1
clone_2
D
DLX1 (log2(TPM+1))
Relative DLX1 expression
LiCl:
(–)
+LiCl
+Wnt3a
DT1_A
DT1_U
n.s.
E
DLX2 (log2(TPM+1))
Relative DLX2 expression
clone_1
clone_2
clone_3
clone_4
H
ADCY2 (log2(TPM+1))
CACNA1C (log2(TPM+1))
CACNA1E (log2(TPM+1))
CACNA2D1 (log2(TPM+1))
GAD2 (log2(TPM+1))
SLC32A1 (log2(TPM+1))
F
PAX6 (log2(TPM+1))
Relative PAX6 expression
G
NEUROG2 (log2(TPM+1))
Relative NEUROG2 expression
I
(–)
+LiCl
DT1_A1
DT1_A2
DT1_A3
DT1_A4
DT1_U1
DT1_U2
DT1_U3
DT1_U4
Nestin EdU Hoechst
J
EdU+/Nestin+ cells (%)
A1(–)
A1+Li
A2(–)
A2+Li
A3(–)
A3+Li
A4(–)
A4+Li
U1(–)
U1+Li
U2(–)
U2+Li
U3(–)
U3+Li
U4(–)
U4+Li

Figure S7| Gene expression analyses and proliferation assay of DT1 iPSC-derived forebrain NSPCs (related to Figure 3)

(A) Heatmap of selected DEGs annotated with Wnt/β-catenin signaling between DT1_A and DT1_U in iPSC-derived 8-day-old NSPCs. Color intensity indicates gene expression level.

(B) Quantification of live cells in 8-day-old NSPCs measured by 7-AAD staining. Live cells were determined as 7-AAD^{-} cells.

(C) EdU incorporation assay showing that there is no difference in the proportion of proliferating live NSPCs between DT1_A and DT1_U on day 8. Cells were stained with anti-CD15 (SSEA1) antibody to analyze neural progenitors only. Data represent mean ± s.e.m. (B and C).

(D) Gene expression levels of *DLX1* analyzed by bulk RNA-seq (left) and RT-qPCR (right) in iPSC-derived 24-day-old NPCs.

(E) Gene expression levels of *DLX2* analyzed by bulk RNA-seq (left) and RT-qPCR (right) in iPSC-derived 24-day-old NPCs.

(F) Gene expression levels of *PAX6* analyzed by bulk RNA-seq (left) and RT-qPCR (right) in iPSC-derived 24-day-old NPCs.

(G) Gene expression levels of *NEUROG2* analyzed by bulk RNA-seq (left) and RT-qPCR (right) in iPSC-derived 24-day-old NPCs. For RT-qPCR, expression values were normalized to *NCAM1* in RT-qPCR; Two-way ANOVA with post hoc *t*-test: $^{*}p < 0.05$; $^{***}p < 0.001$; *n.s.*, not significant ; Wnt3a-treated samples were not subjected to statistical analyses due to the limited sample size (D-G).

(H) Expression levels of selected 'GABA Receptor Signaling'-related genes from bulk RNA-seq of iPSC-derived NPCs on day 24. Two-way ANOVA with post hoc *t*-test: $^{*}p < 0.05$; $^{**}p < 0.01$; *n.s.*, not significant.

(I) Representative images of EdU staining and Nestin immunostaining in 30-day-old NPCs related to Figure 3C. Scale bars, 50 μm.

(J) Quantification of EdU^{+} proliferating NPCs related to Figure 3C. Data from each iPSC clone were presented individually. Bars, mean.

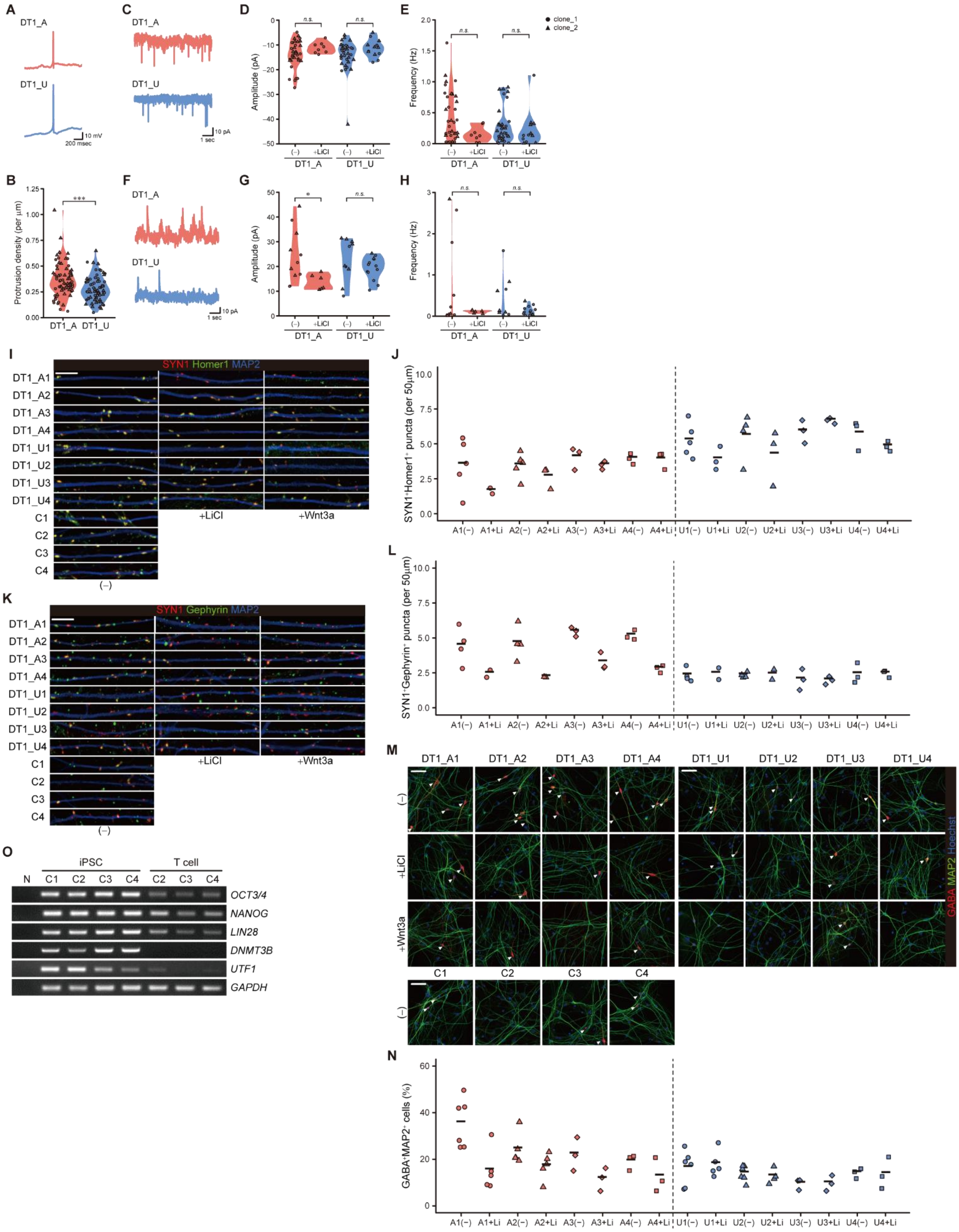

A
DT1_A
DT1_U
10 mV
200 msec
C
DT1_A
DT1_U
10 pA
1 sec
D
Amplitude (pA)
n.s.
n.s.
(−)
+LiCl
DT1_A
DT1_U
E
Frequency (Hz)
clone_1
clone_2
n.s.
n.s.
B
Protrusion density (per μm)
***
DT1_A
DT1_U
F
DT1_A
DT1_U
10 pA
1 sec
G
Amplitude (pA)
*
n.s.
H
Frequency (Hz)
n.s.
n.s.
I
SYN1 Homer1 MAP2
DT1_A1
DT1_A2
DT1_A3
DT1_A4
DT1_U1
DT1_U2
DT1_U3
DT1_U4
C1
C2
C3
C4
(−)
+LiCl
+Wnt3a
J
SYN1⁺Homer1⁺ puncta (per 50μm)
A1(−) A1+Li A2(−) A2+Li A3(−) A3+Li A4(−) A4+Li U1(−) U1+Li U2(−) U2+Li U3(−) U3+Li U4(−) U4+Li
K
SYN1 Gephyrin MAP2
L
SYN1⁺Gephyrin⁺ puncta (per 50μm)
M
DT1_A1 DT1_A2 DT1_A3 DT1_A4 DT1_U1 DT1_U2 DT1_U3 DT1_U4
(−)
+LiCl
+Wnt3a
GABA MAP2 Hoechst
C1 C2 C3 C4
N
GABA⁺MAP2⁺ cells (%)
O
N
iPSC
T cell
C1 C2 C3 C4 C2 C3 C4
OCT3/4
NANOG
LIN28
DNMT3B
UTF1
GAPDH

Figure S8| Functional analyses of iPSC-derived 120-day-old neurons (related to Figure 3)

(A) Sample traces of spontaneous firing of iPSC-derived neurons on day 120.

(B) Quantification of the dendritic protrusion density. Student's *t*-test: $^{***}P < 0.001$.

(C) Sample traces of miniature excitatory postsynaptic currents (mEPSCs) of 120-day-old neurons.

(D and E) Comparison of mEPSC amplitude (D) and frequency (E) between the twins. Each icon corresponds to a value of single cell.

(F) Sample traces of miniature inhibitory postsynaptic currents (mIPSCs) of 120-day-old neurons.

(G and H) Summaries of mIPSC amplitude (G) and frequency (H). Each icon corresponds to a value of single cell. LiCl significantly decreases the amplitude of mIPSCs specifically in DT1_A consistent with the marked reduction of GABAergic synapses and neurons by LiCl treatment during early neurodevelopmental stages (Figures 3G and 3I). Two-way ANOVA with post hoc Student's *t*-test: $^{*}p < 0.05$; *n.s.*, not significant.

(I) Representative images of the immunostaining of excitatory synapses in 120-day-old neurons (related to Figures 3D and 3E). Scale bar, 10 μm.

(J) Quantification of $SYN1^{+}Homer1^{+}$ excitatory synaptic puncta related to Figure 3E. Data from each iPSC clone were presented individually. Bars, mean.

(K) Representative images of the immunostaining of inhibitory synapses in 120-day-old neurons (related to Figures 3F and 3G. Scale bar, 10 μm.

(L) Quantification of $SYN1^{+}Gephyrin^{+}$ inhibitory synaptic puncta related to Figure 3G. Data from each iPSC clone were presented individually. Bars, mean.

(M) Representative images of immunostaining for GABA and MAP2 from 120-day-old neurons (related to Figure 3I; images from DT1_A4, DT1_U4 and C1 are represented in Figure 3H). Scale bars, 50 μm.

(N) Quantification of GABAergic neurons related to Figure 3I. Data from each iPSC clone were presented individually. Bars, mean.

(O) RT-PCR results showing the expression of pluripotent stem cell markers in iPSCs established from control individuals. Parental T cells were used as negative controls; N, no template. C1's T cells were not analyzed due to poor proliferation capacity.

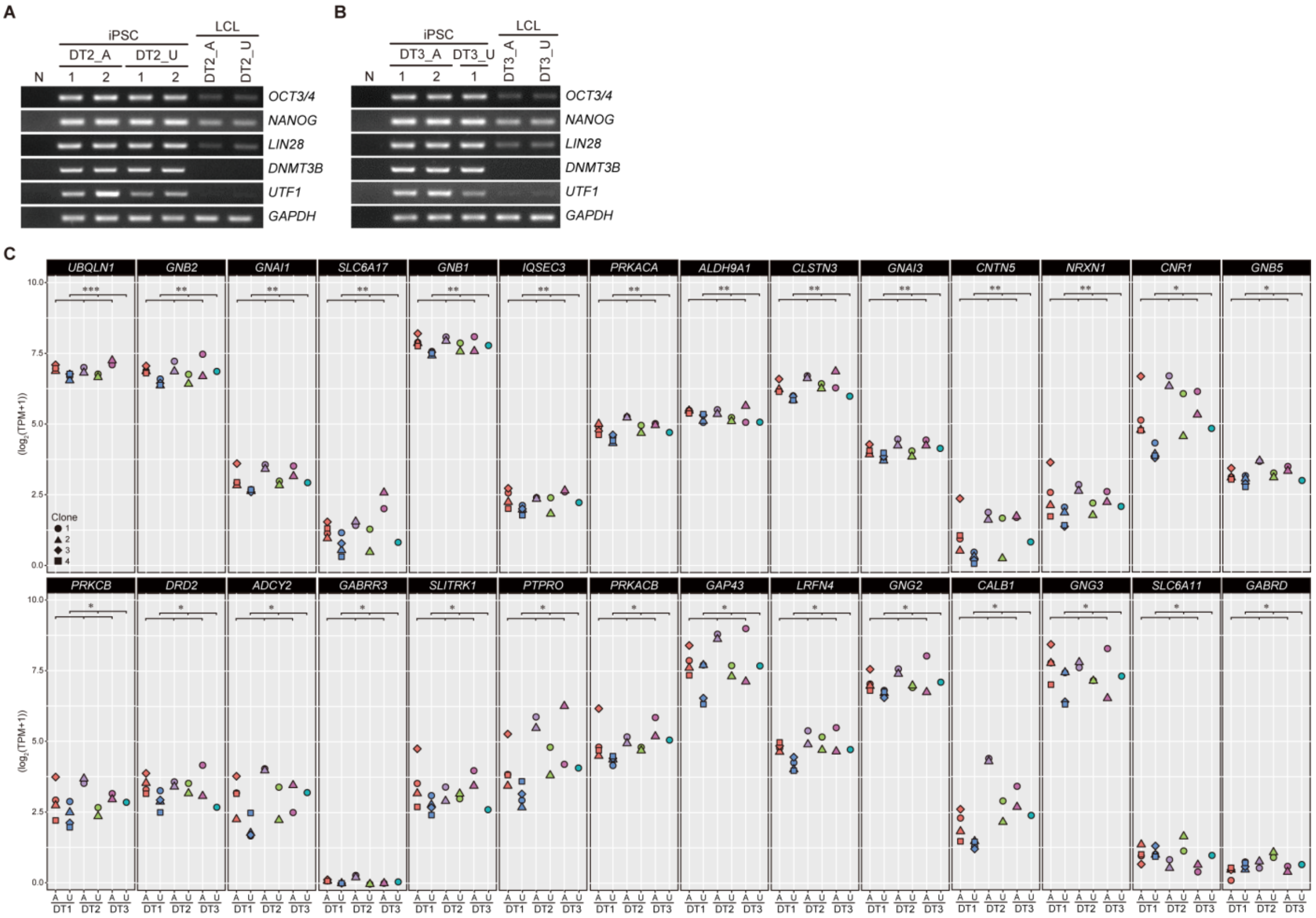

Figure S9| Expression analyses of GABAergic synapse-related genes in 24-day-old NPCs between the psychotic twins and their co-twins (related to Figure 4)

(A and B) RT-PCR results showing the expression of pluripotent stem cell markers in iPSCs established from DT2 (A) and DT3 (B). Parental LCLs were used as negative controls; N, no template.

(C) Gene expression levels of GABAergic synapse-related genes from bulk RNA-seq analysis. A, affected twins; U, unaffected twins. Two-way ANOVA with post hoc *t*-test: $^{*}p < 0.05$; $^{**}p < 0.01$; $^{***}p < 0.001$. Only two genes (*SLC6A11* and *GABRD*) were downregulated in the affected twin-derived NPCs among 146 genes analyzed.

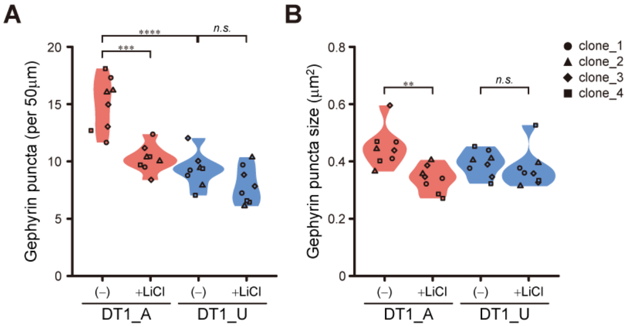

Figure S10| Quantification of inhibitory postsynaptic puncta in iPSC-derived 120-day-old neurons

(A) Quantification of the number of Gephyrin puncta. Two-way ANOVA with post hoc Student's *t*-test: $^{***}p < 0.001$; $^{****}p < 0.0001$; *n.s.*, not significant.
(B) Quantification of the size of Gephyrin puncta. Two-way ANOVA with post hoc Student's *t*-test: $^{**}p < 0.01$; *n.s.*, not significant.

A

Control	Race	N	% Gender	% Total	Age (average)	RIN (average)
Male		146		71.22	45.36	8.36
	AA	67	45.89			
	AS	6	4.11			
	CAUC	68	46.58			
	HISP	5	3.42			
Female		59		28.78	48.22	8.08
	AA	42	71.19			
	AS	1	1.69			
	CAUC	15	25.42			
	HISP	1	1.69			
Total		205			46.18	8.28
	AA	109		53.17		
	AS	7		3.41		
	CAUC	83		40.49		
	HISP	6		2.93		
**Schizo**	**Race**	**N**	**% Gender**	**% Total**	**Age (average)**	**RIN (average)**
Male		114		63.33	47.31	8.06
	AA	49	42.98			
	AS	2	1.75			
	CAUC	60	52.63			
	HISP	3	2.63			
Female		66		36.67	55.16	7.76
	AA	30	45.45			
	AS	1	1.52			
	CAUC	34	51.52			
	HISP	1	1.52			
Total		180			50.19	7.95
	AA	79		43.89		
	AS	3		1.67		
	CAUC	94		52.22		
	HISP	4		2.22		

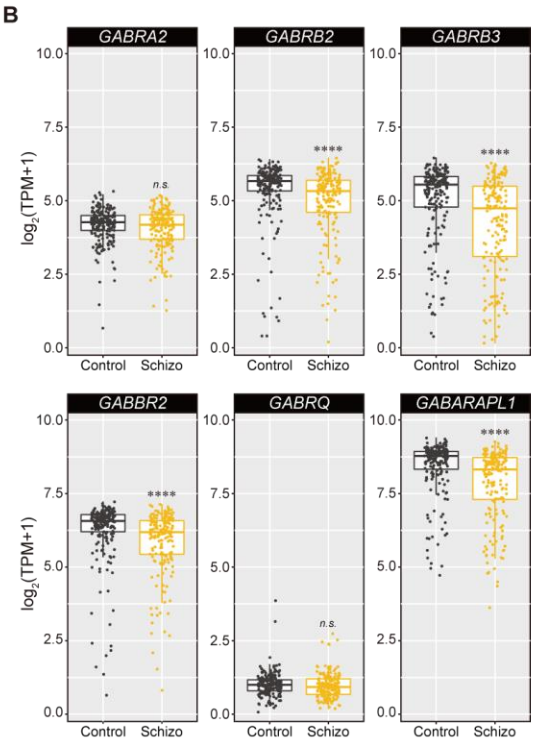

Figure S11| Expression analyses of GABAergic synapse-related genes in postmortem brain tissues between the patients with psychoses and the non-psychiatric control individuals

(A) Summary of postmortem brain samples. AA, African American; AS, Asian; CAUC, Caucasian; HISP, Hispanic; RIN, RNA integrity number.

(B) Representative gene expression levels of GABAergic synapse-related genes from postmortem DPLFC RNA-seq analysis. Schizo, schizophrenia + schizoaffective disorder. Mann Whitney test: $^{****}p < 0.0001$; *n.s.*, not significant.

Supplementary References

72. Agoston N, Li A, Haslinger A, Wizenmann D. Schulte, Genetic and physical interaction of Meis2, Pax3 and Pax7 during dorsal midbrain development. *BMC Dev Biol* 2012; **12**: 10.

73. Rapacioli M, Palma V, Flores V. Morphogenetic and Histogenetic Roles of the Temporal-Spatial Organization of Cell Proliferation in the Vertebrate Corticogenesis as Revealed by Inter-specific Analyses of the Optic Tectum Cortex Development. *Front Cell Neurosci* 2016; **10**: 67.

74. Shcheglovitov A, Shcheglovitova O, Yazawa M, Portmann T, Shu R, Sebastiano V et al. SHANK3 and IGF1 restore synaptic deficits in neurons from 22q13 deletion syndrome patients. *Nature* 2013; **503**: 267-271.

75. Takahashi K, Tanabe K, Ohnuki M, Narita M, Ichisaka T, Tomoda K et al. Induction of pluripotent stem cells from adult human fibroblasts by defined factors. *Cell* 2007; **131**: 861-872.

76. Yu J, Hu K, Smuga-Otto K, Tian S, Stewart R et al. Human induced pluripotent stem cells free of vector and transgene sequences. *Science* 2009; **324**: 797-801.

77. Park IH, Arora N, Huo H, Maherali N, Ahfeldt T, Shimamura A et al. Disease-specific induced pluripotent stem cells. *Cell* 2008; **134**: 877-886.

78. Li S, Xue H, Wu J, Rao MS, Kim DH, Deng W et al. Human Induced Pluripotent Stem Cell NEUROG2 Dual Knockin Reporter Lines Generated by the CRISPR/Cas9 System. *Stem Cells Dev* 2015; **24**: 2925-2942.

79. Espuny-Camacho I, Michelsen KA, Gall D, Linaro D, Hasche A, Bonnefont J et al. Pyramidal neurons derived from human pluripotent stem cells integrate efficiently into mouse brain circuits in vivo. *Neuron* 2013; **77**: 440-456.
